# Exploratory single-cell multiomics suggests naive CD4^+^ T cell dysregulation in treatment refractory rheumatoid arthritis

**DOI:** 10.1016/j.isci.2026.117949

**Published:** 2026-10-27

**Authors:** Samantha L. Smith, Stefano Rossi, Rudresh Shukla, Chuan Fu Yap, Nisha Nair, Jake Butler, Yvonne Tan, Amirah Binti Mohammad Ariff, Ilinca Lazar, Khadijah Patel, Stephen Eyre, Maya H. Buch, Paul Martin, Gisela Orozco

**Affiliations:** 1 Centre for Genetics and Genomics Versus Arthritis, The University of Manchester, Manchester, UK; 2 NIHR Manchester Biomedical Research Centre, Manchester University NHS Foundation Trust, Manchester Academic Health Science Centre, Manchester, UK

**Keywords:** rheumatoid arthritis, difficult-to-treat, multiomics, single cell, sequencing

## Abstract

Around 20% of patients with rheumatoid arthritis (RA) have disease that remains refractory to multiple targeted treatments, highlighting a major clinical challenge. We determine single-nucleus transcriptional and chromatin landscapes (10x Genomics Epi Multiome) on 14,625 primary CD4^+^ T-cells isolated from treatment-naive early and refractory (defined as per the EULAR definition of difficult-to-treat) patients with RA. We systematically analyze circulating regulatory profiles to reveal potential mechanisms and therapeutic targets that may contribute to the difficult-to-treat phenotype. We highlight disease-stage-specific alterations in naive and Th1-like memory CD4^+^ T-cells. In particular, refractory naive cells show signatures of heightened activation, upregulation of pathways aligning with T cell anergy, diminished regulation at regulatory loci (e.g., FOXP1, ICOS) and blunted stimulation responses; consistent with a more desensitized or exhausted state. Genes such as *LGALS3, ZBTB7B* and *FOXO4* emerge as potential candidate regulators of the difficult-to-treat state. Finally, through RA GWAS integration, we prioritize putative causal variants in cis-regulatory elements.

## Highlights

• Naive and Th1-like memory CD4^+^ T cells show altered regulatory networks in refractory RA
• Multiomics identifies potential therapeutic targets, including *LGALS3, ZBTB7B* and *FOXO4*
• GWAS integration prioritizes putative causal RA risk genes

## Graphical abstract

**Figure:**
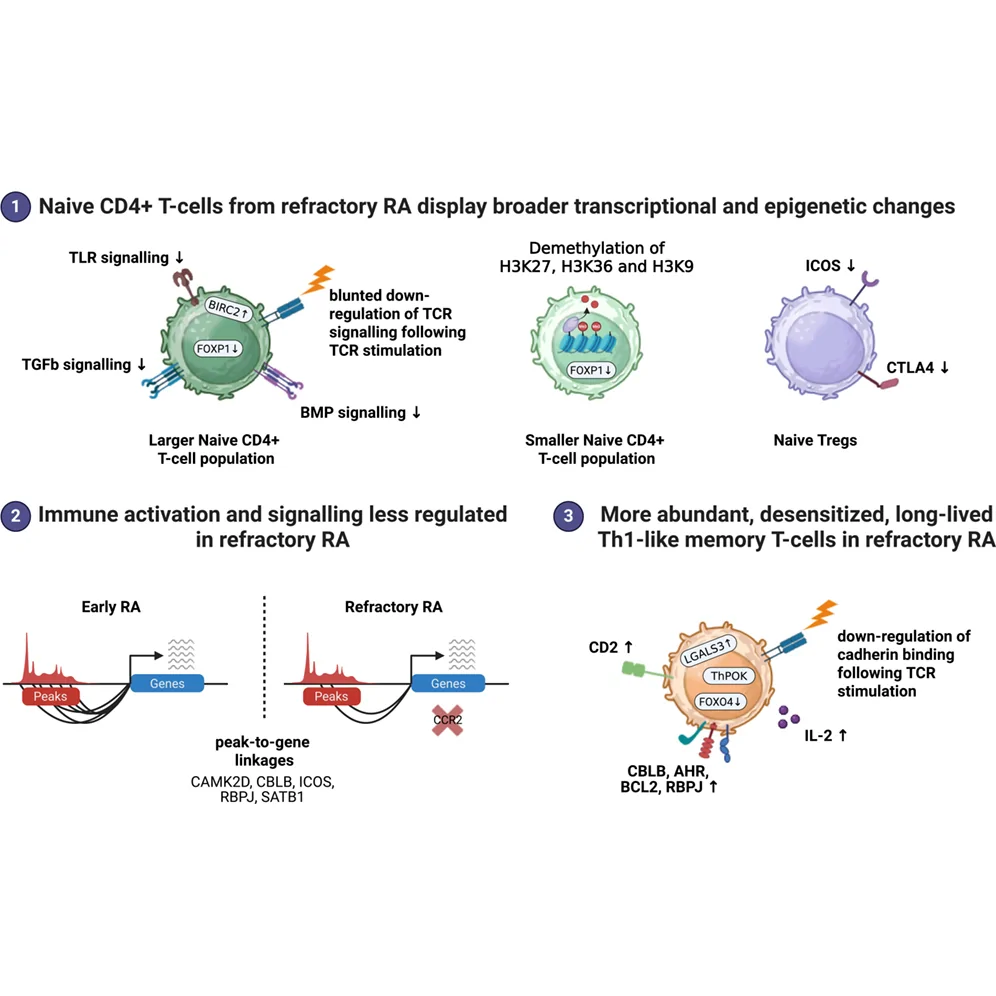

## Teaser

Immunology; Transcriptomics

## Introduction

Rheumatoid arthritis (RA) is a common chronic inflammatory disorder, with an incidence rate ranging from 0.5 to 1% and imposes a substantial personal and societal burden.^1^ It is characterized by joint inflammation that causes pain, swelling, and disability if sub-optimally controlled. Disease-modifying antirheumatic drugs (DMARDs) are employed to reduce inflammation and limit tissue damage.^1^ Biologic and targeted synthetic DMARDs have transformed clinical outcomes, yet treatment often relies on a trial-and-error approach, where patients may cycle through multiple drugs before finding an effective treatment option. Up to 20% of patients will still fail to benefit even after cycling through therapies with different mechanisms of action, a group defined as difficult-to-treat (D2T) RA by the European Alliance of Associations for Rheumatology (EULAR), and we term here as refractory RA (rRA).^2^

Genetic studies have extensively implicated T cell dysregulation as one of the main drivers of RA etiopathology. Genome-wide association studies (GWASs) have mapped over 100 RA susceptibility loci, with most of these variants being identified within enhancer and promotor regions of CD4^+^ T-cells.^3^^,^^4^^,^^5^^,^^6^ Capture Hi-C studies have shown that RA risk enhancers engage with distal genes through long-range interactions in T-cells.^7^^,^^8^ Expression quantitative trait locus (eQTL) analyses have further illuminated RA regulatory networks by linking genetic variants to gene expression and have revealed cell- and state-specific effects across CD4^+^ T cell subsets upon stimulation; granularity which is lost in bulk or steady-state analyses.^9^^,^^10^^,^^11^

While multiple studies have investigated clinical predictors of rRA,^2^ few studies have investigated the underlying biological mechanisms. Yang and colleagues reported disrupted immunity in D2T compared to healthy volunteers and early or treated patients with RA, documenting reduced absolute numbers of regulatory T-cells (Tregs) and a higher ratio of T-helper (Th)17/Treg cells.^12^ Interestingly, low-dose IL-2 recovered Treg numbers and preliminary data suggest this could be a viable treatment option for D2T RA.^12^ Furthermore, gene expression profiles of Th17 cells have shown increased expression of *USF2*, a transcription factor (TF) enforcing proinflammatory programs, in rRA patients compared to anti-tumor necrosis factor (TNF) responders.^13^

Causality is therefore established through cell type and state-resolved genomic assays that bridge risk variants to genes, and these data implicate CD4^+^ T cell imbalance as a potential basis for refractory disease and a potential therapeutic target. Enhancer-gene wiring has been achieved with multi-omics approaches across multiple tissues and cell types by large consortia such as ENCODE and BLUEPRINT, which provide orthogonal support to infer causal relationships between non-coding DNA segments and disease susceptibility.^14^ However, these efforts largely focus on healthy or bulk cell populations and have not yet resolved cell state- and disease stage-specific regulatory interactions.

Despite the well-established role of CD4^+^ T-cells in RA etiopathogenesis,^15^ exploring their regulatory programmes and stimulation-dependent responses in rRA has not yet been described. We hypothesize that rRA is underpinned by CD4^+^ T cell dysregulation, which can be characterized through multi-omics analyses. We report on single-nucleus RNA-sequencing (snRNA-seq) and single-nucleus ATAC sequencing (snATAC-seq) from CD4^+^ T-cells isolated from treatment-naive early RA (eRA) and patients with rRA, enabling the simultaneous measurement of chromatin accessibility (gene regulation) and gene expression on the same cells. This approach enabled a comparative framework of subset-specific immunopathology, comparing rRA with eRA—two opposing ends of the RA disease continuum—to explore mechanisms potentially associated with treatment-refractory disease.

## Results

### Circulating CD4^+^ T cell heterogeneity of patients with rheumatoid arthritis

To profile transcriptional and chromatin landscapes, snRNA-seq and snATAC-seq libraries were generated on CD4^+^ T-cells isolated from three treatment-naive eRA and three patients with rRA (Table 1), using the 10x Genomics Epi Multiome ATAC + Gene Expression pipeline according to the manufacturer’s instructions following pooling of samples (Figure 1A). The cohort had a median age at recruitment of 52 (IQR 52–62) years in the eRA group and 61 (IQR 57.5–63) in the rRA group. The average disease duration was 2 years for the eRA group and 32.7 years for the rRA group, respectively. All patients had active disease, defined as a disease activity score as measured in 28 joints (DAS28-ESR) >3.2, and none of the patients were currently receiving methotrexate.

**Table 1:** Baseline characteristics of patients enrolled within the study

Participant	RA stage	Sex	RF/ACPA	DAS28-ESR	On steroid	On b/tsDMARD	No. previous b/tsDMARD
1	eRA	F	+/+	5.75	no	no	0
2	eRA	F	+/+	5.33	no	no	0
3	eRA	M	+/−	3.27	no	no	0
4	rRA	M	+/−	7.01	yes∗	baricitinib	8
5	rRA	F	+/+	5.77	no	rituximab	3
6	rRA	M	+/−	4.88	no	no	4

eRA = early RA, rRA = refractory RA, RF = rheumatoid factor, ACPA = anti-citrullinated protein antibodies; DAS28-ESR = disease activity score in 28-joints; ESR = erythrocyte sedimentation rate; MTX = methotrexate; b/tsDMARD = biological/targeted synthetic disease modifying anti-rheumatic drugs; F = female; M = male. ∗10 mg daily oral prednisolone.

**Figure 1:**
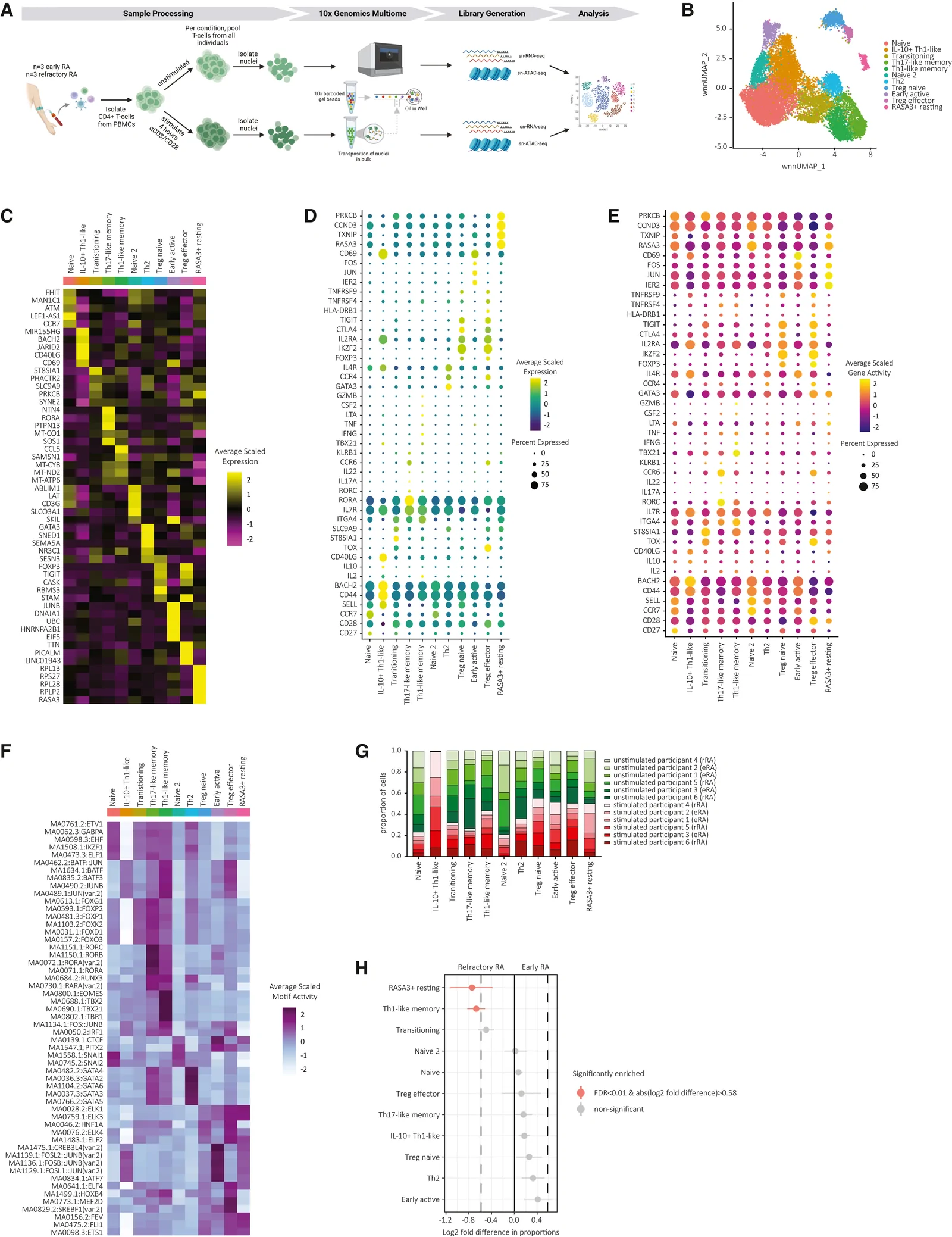
*Circulating CD4*^*+*^*T cell heterogeneity of* patients with *rheumatoid arthritis* (A) Schematic showing study workflow. Peripheral blood samples were collected from patients with treatment-naive early (*n* = 3) and treatment-refractory (*n* = 3) RA. CD4^+^ T-cells were isolated and left unstimulated or stimulated with αCD3/CD28 beads for 4-h and analyzed using single-nucleus RNA-sequencing (10x Genomics Chromium). Created in BioRender. Smith, S. (https://BioRender.com/8khpr7l) is licensed under CC BY 4.0. (B) Integrated UMAP of all cells passing quality control, colored by annotated populations. (C) scRNA gene expression heatmap for the top 5 markers distinguishing each population. Populations plotted in columns and genes in rows. Gene expression is color-coded using average scaled expression values per population, based on a *Z* score distribution, ranging from low (purple) to high expression (yellow). (D) scRNA gene expression for selected canonical T cell markers. The size of the dot represents the percentage of cells expressing the markers, and the color encodes the average scaled gene expression values, ranging from low (blue) to high expression (yellow). (E) scATAC gene activity scores for the markers shown in D. The color encodes the average scaled gene activity values, ranging from low (purple) to high activity (yellow). (F) scATAC motif activity heatmap for the top 5 differentially active motifs within each population. Populations are plotted in columns and motifs are shown in rows. Motif activity is color -using average scaled activity values per population, based on a *Z* score distribution, ranging from low (light blue) to high activity (dark purple). (G) Fraction of each sample comprising each CD4^+^ T cell population. Unstimulated samples are shown in green and stimulated cells in red. (H) Comparative analysis of proportions of CD4^+^ T cell populations across disease stage. Significantly enriched populations (>0.58 log2 fold difference and FDR < 0.01 (Fisher’s exact test, 10,000 permutations)) are shown in red, and non-significant comparisons are shown in grey. No significant enrichment was observed for eRA.

Following quality control, a total of 14,625 single nuclei (7,997 unstimulated and 6,628 stimulated) were retained for analysis. Weighted nearest neighbor analysis resulted in the unsupervised identification of 11 populations (Figure 1B). Gene expression profiles and gene activity scores of the top markers expressed per population were used for cell annotation (Figures 1C and S2A; Table S1), as well as canonical T cell markers (Figures 1D, 1E, and S2B). Annotation broadly resolved the populations into naive, memory, active, resting, Th2 and Tregs. Within the Th1-like memory compartment, a small cluster of *GZMB-, PRF1-, TNF-, and IFNγ-*expressing cells was detected, consistent with a terminally differentiated effector memory cell (TEMRA) phenotype (Figure S2C); their low abundance likely prevented distinct clustering.

A total of 118,387 open chromatin peaks were identified, most of which (45.8%) overlapped with accessible intergenic regions or were within 3 kb of transcription start sites, assumed to contain putative regulatory elements, such as promoter and enhancer regions (Figure S2D). Populations were significantly enriched for lineage- or phenotype-determining TF motifs (Figure 1F). We observed enrichment of known T cell lineage TFs, *RORC*, *TBX21*, and *GATA3*, which further supports our annotations, whilst enrichment of *MEF2D* and *SREBF1* in Treg effector cells aligns with this subtype function.^16^^,^^17^ Similarly, *IRF1* and *RARA* are required for Th1 and sustained Th17 responses, respectively.^18^^,^^19^ However, as expected for a relatively homogeneous population, most TF motifs were not uniquely enriched (Figure S2E).

Populations included cells from all donors, disease stages, and conditions (Figure 1G), except one enriched for stimulated cells displaying an atypical polyfunctional activation state characterized by co-expression of *IFNγ, IL-2*, *TNF,* and high *IL-10* (Figure S2F). This population also expressed *TBX21*, supporting a Th1-lineage identity, consistent with previous reports that activated Th1 cells can transiently acquire IL-10 expression as part of a feedback regulatory program during strong immune activation.^20^ These cells also express CD40LG and ICOS, which are broadly induced upon activation in CD4^+^ T-cells.^21^^,^^22^^,^^23^^,^^24^^,^^25^ IL-21 expression was also observed within this cluster and upon further inspection, we identify a smaller subset of cells expressing high levels of *CXCR5*; however, these cells do not exhibit a full canonical Tfh program (e.g., *BCL6, PDCD1*) nor form a distinct transcriptional cluster, indicating that Tfh-like features are restricted to a minor subpopulation rather than representing a discrete lineage within this cluster (Figure S2G). Consistent with the observation that populations comprised cells from all donors, disease stages, and conditions, variance decomposition analysis identified stimulation status and cell population identity as the major drivers of transcriptional variability across CD4^+^ T-cells, whereas patient-level clinical variables, including serostatus and age, contributed comparatively small to modest effects overall (Figures S2H and S2I).

To assess the influence of disease stage on cell composition, proportions were compared, revealing significant enrichment (log2FC > 0.58 and FDR < 0.01) of Th1-like memory and RASA3^+^ resting cells in rRA (Figure 1H). As most populations did not differ, the analysis next focused on whether gene expression profiles did (Figure S3). No significant differences were observed within the IL-10^+^ Th1-like, Th2, and Treg populations, nor in RASA3^+^ resting cells (FDR < 0.05). Across the remaining populations, significantly more genes were upregulated in rRA (absLog2FC > 0.585 and FDR < 0.05) (Table S2). Interestingly, within the larger naive CD4^+^ T cell population, we observed upregulation of genes involved in actin binding and negative regulation of cell fate decision pathways. Conversely, the smaller naive CD4^+^ T cell population, i.e., naive 2 on the uniform manifold approximation and projection (UMAP), displayed enrichment of histone demethylation, suggestive of large-scale chromatin remodeling and a pre-activated or poised phenotype.

### Defining the regulatory blueprint: Cis-elements and key gene controllers

Candidate cis-regulatory elements (CREs) were identified by correlating chromatin accessibility peaks with gene expression. Using the full dataset, 12,516 peak-to-gene linkages were identified (Figure S4A). Most peaks (89.4%) were not linked to any gene, reflecting the small effect sizes of typical CREs.^26^ Where links were observed, only 58.2% were linked to their nearest gene, consistent with experimental estimates of enhancer-gene pairing.^27^

In line with previous studies,^28^^,^^29^ a subset of highly regulated genes (HRGs) linked to a large number of peaks were identified (Figure S4B; Table S3). *BACH2* was the most regulated HRG (67 links), a TF that plays a pivotal role in CD4^+^ biology and has been linked to RA susceptibility.^30^^,^^31^ Other notable TFs involved in differentiation and function included IKZF2, ARID5B, MAF, and ZEB2, as well as genes involved in activation and signaling, CAMK2D, KLF6, MYC, and *CBLB*. Next, data were stratified by disease stage and peak-to-gene linkages were recalculated, yielding 14,907 in eRA and 15,002 in rRA (Figure S4C). Disease-stage specific links were observed, suggesting that chromatin accessibility and regulatory interactions differ between disease stages (Figure S4D). A total of 81 and 85 HRGs were identified in eRA and rRA, respectively, comprising 101 unique HRGs (Figures 2A and 2B; Table S3); with 11 showing differential regulation (Figure 2C). In eRA, more peak-to-gene linkages were observed for *CAMK2D*, *CBLB*, *ICOS*, *RBPJ*, *SATB1* and *LRRN3;* genes largely involved in stimulation responses and differentiation. Notably, *SATB1*, key in chromatin organization and CD4^+^ T cell development,^32^ displayed 24 links in eRA versus only 9 in rRA. Differential regulation for less regulated genes was also explored (<20 peak-to-gene or no links in one disease stage and >4 in the other) (Figure S4E), highlighting *CCR2*, with 12 links in eRA and 0 links in rRA; *CCR2* is involved in directing T-cells to inflammation sites, including RA.^33^

**Figure 2:**
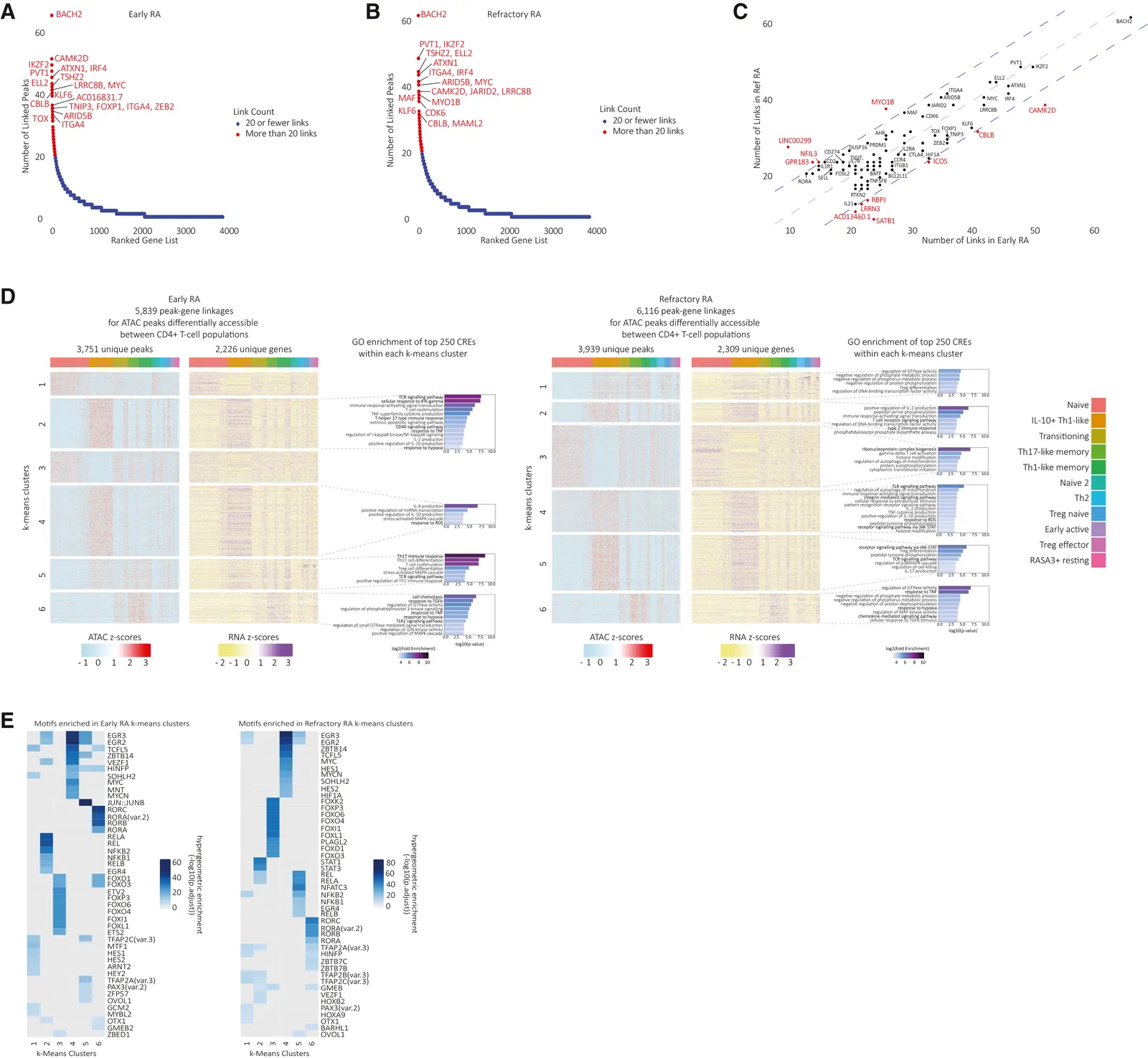
Defining the regulatory blueprint: cis-elements and key gene controllers (A) Genes ranked by the number of peak-to-gene linkages identified for each gene in eRA samples: 81 HRGs had >20 peak-to-gene linkages in eRA. (B) Same as A) but repeated for rRA samples: 85 HRGs had >20 peak-to-gene linkages in rRA. (C) Scatterplot showing the comparison of HRGs identified in eRA (x axis) and rRA (y axis). In total, 101 unique HRGs were identified. The axes show the number of peak-to-gene linkages identified for each gene. Significantly different HRGs were based on residuals from the line of identity and defined as points exceeding ±1.5 standard deviations of the residuals. (D) Heatmaps showing chromatin accessibility (left) and gene expression (right) for peak-to-gene linkages of differentially accessible (DA) ATAC peaks. ATAC peaks were clustered using *k*-means clustering (*k* = 6). GO term enrichment for the top 250 CRE genes (ranked by number of peak-to-gene linkages) for selected *k*-means clusters is shown to the right of the gene expression heatmap. CD4^+^ T cell populations represent columns. Rows represent *k*-means clusters. The eRA group is shown on the left and the rRA group is shown on the right. (E) Hypergeometric enrichment of TF motifs enriched in peaks from each of the k-means clusters from D. The eRA group is shown on the left and the rRA group is shown on the right.

Population specificity of HRG expression was assessed next. Besides known markers such as *IKZF2*, *TIGIT,* and *RTKN2* in Tregs, most HRGs were broadly expressed across all populations. Disease-stage differences within populations were subsequently assessed, revealing subtle but notable changes in genes such as *ICOS* and *FOXP1*. In rRA, fewer peak-to-gene linkages to *FOXP1* and reduced gene expression were observed in both naive CD4^+^ T cell populations. Additionally, fewer peak-to-gene linkages to *ICOS* and lower gene expression in naive Tregs from patients with rRA were observed; *ICOS* was previously identified as significantly differentially regulated (Figure S4F). *FOXP1* maintains T cell quiescence, keeping cells within a naive state,^34^ whilst *ICOS* supports the survival and suppressive function of Tregs.^35^

### Regulatory hubs: Cis-elements and their gene targets

To explore global regulatory heterogeneity of CREs in CD4^+^ T-cells by disease stage, we first filtered for peaks linked to genes and then clustered based on DA peaks between the CD4^+^ T cell populations using k-means clustering. This revealed population-specific regulatory modules enriched for distinct TF motifs (Figures 2D and 2E). For example, module 6 in both eRA and rRA is enriched in memory populations and shows activation of pathways such as TGF-β, TNF signaling, and metabolic processes supporting long-term survival and re-activation. In contrast, modules 5 and 2 in eRA and 5 and 1 in rRA are not enriched in naive CD4^+^ T cell populations, instead showing GO terms related to co-stimulation, T cell receptor (TCR) signaling and differentiation, consistent with activated or specialized effector-like states. IL-10^+^ Th1-like cells show module enrichment for pathways linked to cytokine production and key signaling cascades (e.g., TCR, NF-κB, JAK-STAT, CD40), highlighting the diverse and immediate responses that are initiated upon activation. Of note, reflecting a homogeneous population, most regulatory modules were enriched for ontologies linked to differentiation, activation, proliferation, adhesion, cytokine production, and inflammation (Figure S4G).

As expected, based on the similarity in population proportions, similar global regulatory patterns were observed between disease stages. However, consistent with the enhancer analyses where we observed disease stage-specific links and subtle shifts in gene expression, specific changes within these shared frameworks were also observed, with rRA showing a greater number of enriched biological pathways across all modules (Figure 2D). In terms of uniquely regulated processes, 19 and 23 pathways were uniquely enriched in eRA and rRA, respectively. In eRA, these pathways were primarily concentrated within two modules and emphasize processes initiating immune responses, including T cell activation, γδ T cell differentiation, and IFNγ and CD40 signaling. In contrast, pathways unique to rRA were distributed across multiple modules and reflected a shift toward managing chronic inflammation and attempts at regulatory control. These included positive regulation of cytokine production, negative regulation of protein phosphorylation, increased protein synthesis, and metabolic shifts that hint at altered energy balance and intracellular signaling that may help maintain the chronic inflammatory environment. Similarly, motif analysis revealed comparable patterns of TF enrichment within modules across disease stages. Notably, *ZBTB7B* (*ThPOK*) was uniquely enriched in rRA module 6 (Figure 2E); this TF is known to repress pathways associated with short-lived or exhausted effector T cell states.^36^

### Differential TF activities and putative target genes

To identify active transcriptional regulators across all populations and disease stages, we next correlated TF gene expression with motif activity (Figure 3A). We identified 19 active TFs (correlation > 0.5), of which 14 were high-confidence after filtering low-expression genes. Six TFs showed increased activity in rRA (*BACH1, CREM, FOSL1, SMAD2, RUNX2, STAT1*). Most were active across all populations, with *BACH1* and *FOSL1* displaying greater population specificity—absent in naive, early active, or resting cells (Figure 3B).

**Figure 3:**
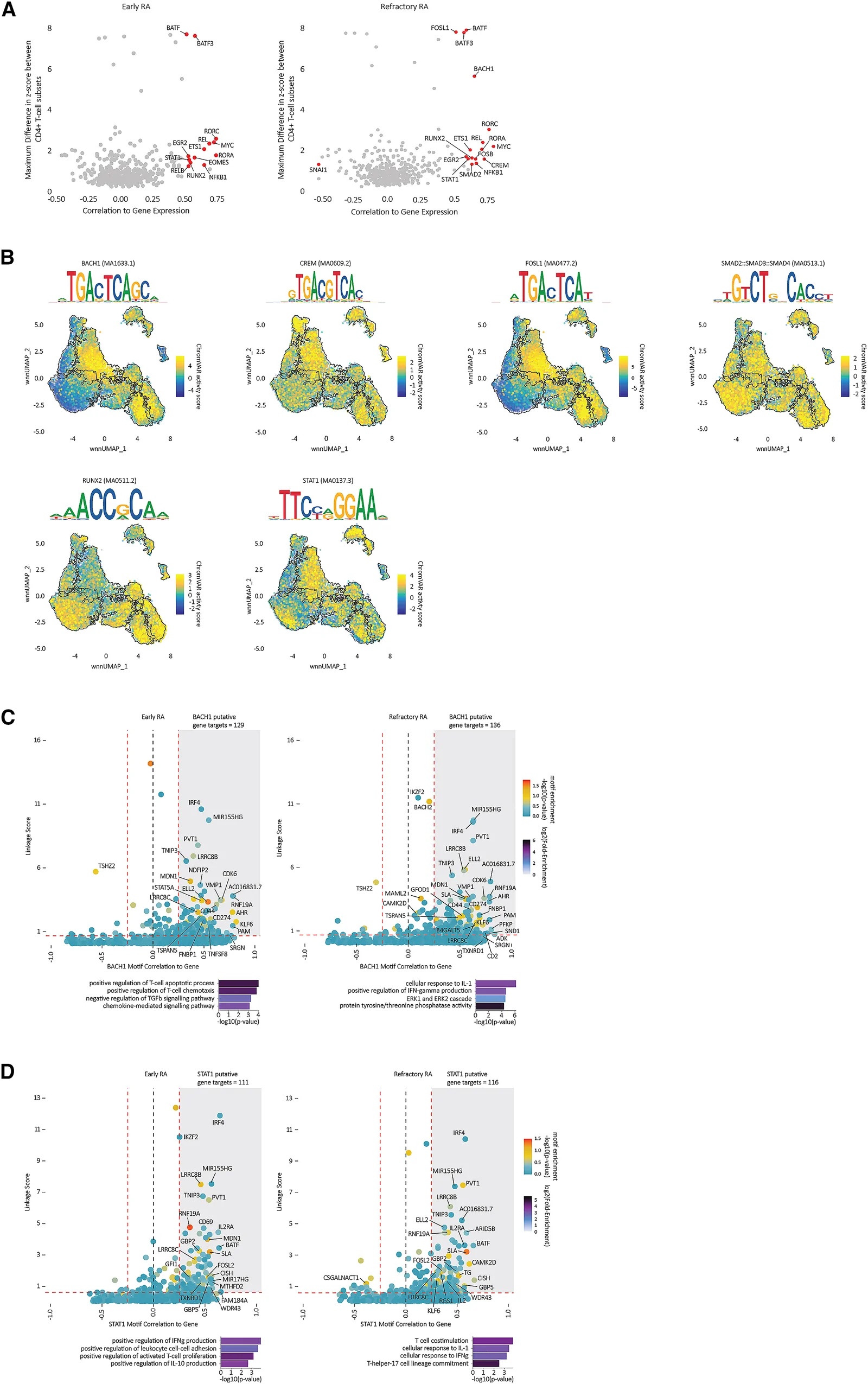
Differential TF activities and putative target genes (A) Plot showing the correlation between TF motif activity and corresponding TF gene expression across CD4^+^ T cell populations (x axis) against the maximum difference in chromVAR deviation *Z* score between populations (y axis). “Active” TFs were defined as TF’s with a maximum chromVAR difference in the top quartile and a Pearson’s correlation greater than 0.5 and are colored in red. The eRA group is on the left and rRA on the right. (B) Plots showing per-cell chromVAR motif activities for TFs of interest using all cells. Each point represents a single cell, positioned according to the UMAP dimensions. Color intensity reflects motif activity as quantified by chromVAR, with warmer colors indicating higher activity. Cells are ordered such that those with higher expression levels are plotted on top to improve visualization of positively expressing populations. Position weight matrices corresponding to each motif are displayed above the plots. (C) Identification of putative gene targets for BACH1 in eRA (left) and rRA (right). The x axis shows the Pearson’s correlation between the TF motif activity and gene expression for all expressed genes across CD4^+^ T cell populations. The y axis shows the TF LS (for all linked peaks, sum of motif score scaled by linkage correlation). Color of points indicates the hypergeometric enrichment of the TF motif in all linked peaks for each gene. Putative gene targets are indicated in the shaded area (motif correlation to gene expression > 0.25, LS > 75th percentile). GO term enrichments for the gene targets are shown. (D) Same as in C but for *STAT1*.

Given the subtle differences in gene regulation observed between disease stages, it was therefore presumed that differential TF activity regulates distinct gene sets. To test this, the motif activity of each of the six TFs was correlated with the expression of all expressed genes and a linkage score was computed as described by Ober-Reynolds et al.^37^ (Table S4). Overall, these TFs mainly function as activators, showing positive correlation with gene expression, except *RUNX2*, which displays both activating and repressor properties (Figures 3C, 3D, and S5A–S5D).

This analysis suggests a dynamic shift in transcriptional regulation across disease stages, from balanced immune modulation in eRA to reinforcement of chronic inflammation in rRA, highlighting candidate regulatory programs which may warrant further investigation in the context of treatment-refractory disease; *BACH1* and *STAT1* exemplify this transition. In eRA, we show that *BACH1* (Figure 3C) limits prolonged T cell activation and regulates apoptosis, while suppressing TGF-β signaling, which are consistent with early immune activation and inflammatory priming. In contrast, in rRA, *BACH1* regulates genes involved in IL-1 and IFN-γ responses, ERK signaling and phosphatase activity, supporting a role in sustaining chronic inflammation. Similarly, *STAT1*, which plays both pro-inflammatory and regulatory roles in immune responses,^38^^,^^39^ also shifts functionally (Figure 3D). In eRA, we found *STAT1* to be associated with a balanced transcriptional program, promoting IFN-γ and IL-10 signaling.^40^ While in rRA, its activity becomes increasingly upregulated and skewed toward pro-inflammatory gene expression, upregulating genes involved in co-stimulation, IL-1/IFN-γ responsiveness and Th17 commitment; an observation which is consistent with a transition toward pathogenic inflammation as previously observed.^35^

To explore the therapeutic relevance of these transcriptional shifts, genes uniquely targeted by these six TFs in each disease stage were examined for potential drug targets by querying the Open Targets database.^41^ Ten drug-targeted genes were identified as unique to eRA, and fourteen to rRA (Table S5). Although predicted upregulation did not always match gene expression levels (Figures S5E and S5F), genes such as *LGALS3* showed both unique targeting and higher expression in select rRA populations (Figure S5F).

### Differential responses to stimulation

We next examined how T cell activation changes differ between eRA and rRA under heightened inflammatory conditions, excluding the predominantly stimulated IL-10^+^ Th1-like population. Across all populations, 4,394 unique genes responded to stimulation (*p* value < 0.05), with naive CD4^+^ T-cells showing the greatest responsiveness and the quiescent RASA3^+^ resting population the least (Figure S6A). Th1-like memory cells showed the greatest number of unique changes (Figure S6B). Notably, the two naive CD4^+^ T cell populations differed, with the smaller naive 2 population displaying reduced responsiveness to stimulation.

We next assessed whether stimulation responses varied by disease stage. Four populations (the larger naive, transitioning, Th1-like and Th17-like memory cells) showed significant differences (adjusted *p* value < 0.05), but only the larger naive and Th1-like populations had sufficient gene numbers (138 and 105, respectively) to support reliable GO analysis and were therefore the focus of further investigation (Figures 4A, 4B, and S6C; Table S6).

In naive CD4^+^ T-cells (Figure 4A), we identified a cluster of genes with comparable baseline expression across disease stages that decreased following stimulation, though less so in rRA. These genes regulate immune synapse formation, membrane protein localization, TCR signaling, actin remodeling, and T-cell proliferation. Additionally, we found *BIRC2*, an anti-apoptotic factor, was elevated pre-stimulation in rRA. In Th1-like memory cells (Figure 4B), one gene cluster increased following stimulation in eRA but decreased in rRA; these genes are linked to cadherin binding (important for T-cell-antigen-presenting cell interactions and immune synapse stability) and the *JNK* stress pathway, reflecting adaptive activation in eRA but possible desensitization in rRA. Another cluster showed similar stimulation responses but higher baseline expression in rRA, involving GO terms including TNF signaling, ER stress, and T-cell anergy, suggesting chronic activation and attempted, but insufficient, immune regulation.

**Figure 4:**
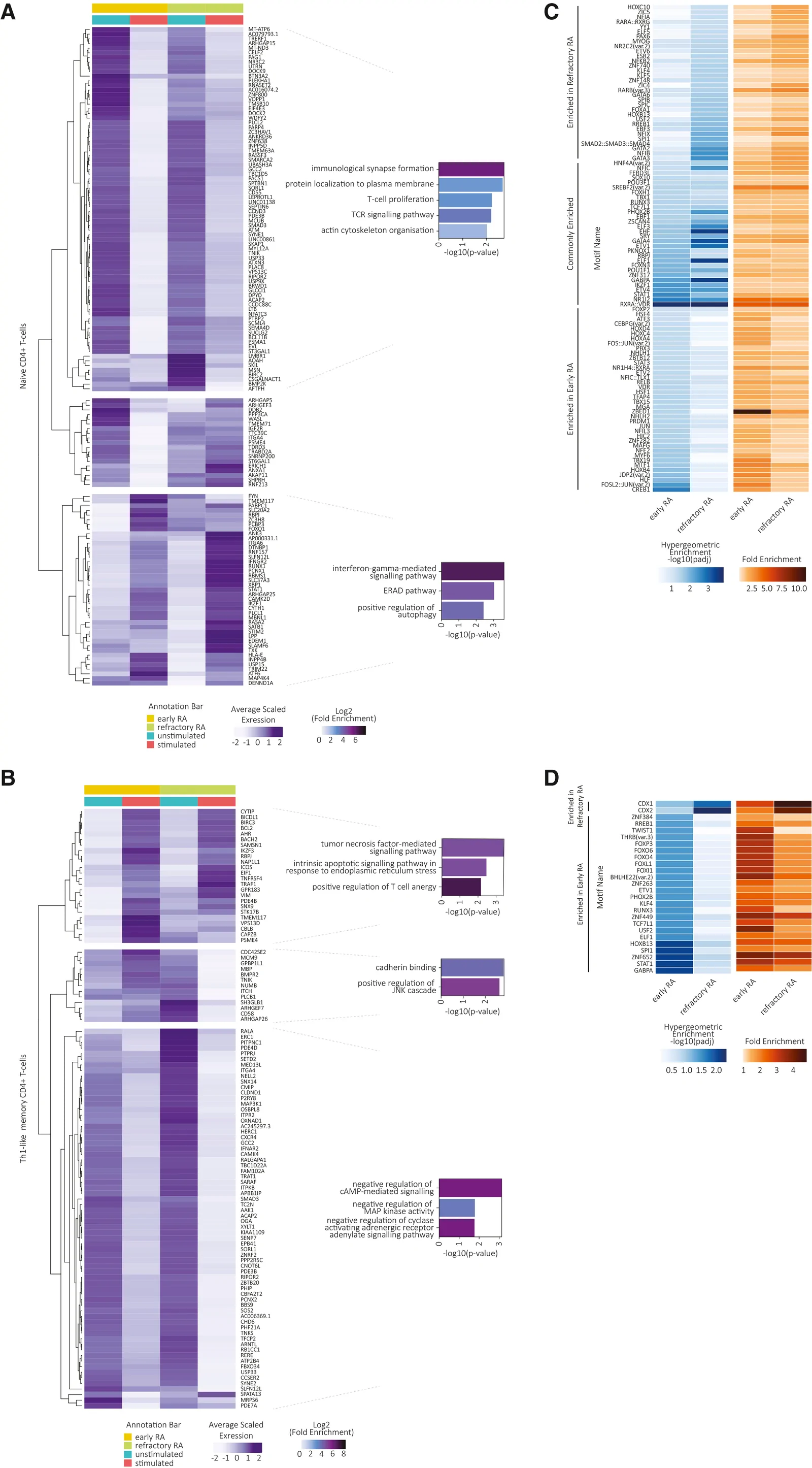
Differential responses to stimulation (A) Heatmap showing gene expression for genes differentially responding to stimulation in early and rRA cases in Naive CD4^+^ T-cells. 138 genes were identified as differentially responding. Columns show average scaled expression values by disease stage (yellow for eRA and green for rRA) and by disease stage further split by unstimulated (blue) and stimulated (red) samples. Genes are shown in rows and were clustered using *k*-means clustering. Gene expression is color coded using average scaled expression values, based on a *Z* score distribution, ranging from low expression (white) to high expression (purple). GO term enrichment was performed by *k*-means cluster. (B) Same as in A but for Th1-like memory CD4^+^ T-cells where 105 genes differentially respond to stimulation between disease stages. (C) Heatmaps showing motif enrichment (blue) and fold enrichment (orange) by disease stage in Naive CD4^+^ T cell stimulation responsive peaks (peaks differentially responding to stimulation and linked to genes differentially responding to stimulation using the peak-to-gene linkages). 68 motifs were found to be enriched in eRA and 62 in rRA (adjusted *p* value < 0.05). Motifs enriched in eRA are shown towards the bottom, those enriched in refractory are towards the top, and motifs commonly enriched are in between. (D) Same as in C but for motifs enriched in Th1-like memory stimulation responsive peaks. 24 motifs were found to be enriched in eRA and 2 in rRA.

To investigate the regulatory basis of these transcriptional differences, we linked differentially responsive genes to corresponding DA chromatin regions using the peak-to-gene linkages. In naive CD4^+^ T-cells, eRA and rRA showed similar numbers of stimulation-responsive peaks (56 and 53), with a slight bias toward increased accessibility in rRA (58% vs. 54%). In Th1-like memory cells, eRA had 32 peaks (40% increased), while rRA had 25 (60% increased), highlighting an inverse trend between stages. In both populations, these stimulation-responsive peaks were mainly present within intronic and intergenic regions, emphasizing the role of distal regulatory elements in stimulation (Figure S6D). Motif enrichment of stimulation-responsive peaks revealed key TFs that may be driving these gene expression differences between disease stages. In naive CD4^+^ T-cells from eRA, *STAT3*, STAT1, *FOS::JUN*, *CREB1* and *ATF3* motifs were enriched (Figure 4C), whilst in Th1-like memory cells, eRA showed greater enrichment of *RUNX3*, *STAT1*, *FOXO4*, *KLF4*, and *FOXP3* motifs (Figure 4D). To further link TF activity with the observed transcriptional responses to stimulation, we correlated chromVAR-derived TF activities with a single-cell gene program, derived using the genes that show differential responses to stimulation between disease stages (Figure S6E). While many TFs showed similar associations across disease stages, several displayed disease-specific relationships with the response program. Notably, in line with the motif enrichment analysis, STAT1 and STAT3 activity showed stronger negative correlations with the stimulation-response program in eRA naive CD4^+^ T-cells compared to rRA, suggesting stronger coupling between STAT-associated regulatory activity and downstream transcriptional responses in early disease.

### Functional annotation of RA risk variants

Finally, to refine the genetic basis of RA, CREs were examined in relation to GWAS variants using a consensus peak-to-gene linkage set, which was created by integrating all previously identified links with new linkages identified in unstimulated and stimulated CD4^+^ T-cells. This resulted in 26,345 unique connections (Figure S7A). As expected, we observed a significant increase in linkages following stimulation, underscoring the context-specific nature of regulatory interactions.

Lead SNPs from the 2022 multi-ancestry RA GWAS,^42^ along with SNPs in high LD (*R*^2^ > 0.8) were compiled as previously described,^43^ resulting in a total of 2,818 variants. Of these, a total of 87 SNPs overlapped with a CRE (Table S7). CREs were significantly enriched for RA GWAS variants (OR 1.59, *p* value < 0.001), indicating that CD4^+^ T cell regulatory elements identified in this study overlap disease-associated loci more than expected by chance. To further quantify the contribution of these regulatory elements to RA genetic architecture, we applied stratified LD score regression (LDSC) conditioned on the baselineLD v2.2 functional annotation model, which allows estimation of the contribution of CD4^+^ T cell regulatory elements to RA SNP heritability beyond a comprehensive set of established functional genomic annotations. Using this model, the total SNP heritability of the trait was estimated at 0.147 (14.7%, SE 0.012); consistent with prior estimates.^44^ Within this joint model, CD4^+^ T cell regulatory annotations contributed significantly to RA heritability, with stimulated CREs accounting for 16.2% of SNP heritability and unstimulated CREs accounting for 7.4%; consistent with stronger activation-dependent genetic enrichment in stimulated cells.

On average, a CRE-linked peak contained 1.3 GWAS SNPs and linked to 1.61 genes (Figures S7B and S7C). Together, these variants spanned 32 loci, representing 20.2% of all RA GWAS loci, highlighting the key role of CD4^+^ T-cells in RA pathogenesis. Per GWAS locus, an average of 2.72 SNPs were found within 2.09 CRE-linked peaks (Figures S7D and S7E). Only 7 lead GWAS SNPs overlapped a CRE; 4 were solitary, and 3 had additional SNPs in LD. In our dataset, variant-gene assignments often differed from proximity-based GWAS annotation. Among the 32 loci analyzed, 19 were linked to their proximity-based gene, but only 6 exclusively so. Most loci showed additional connections, revealing known, novel, and context-specific regulatory relationships (Table S7).

Consistent with previous CRISPRi-based studies,^45^ which revealed shared CREs regulating *CTLA4* and *ICOS* not detected by conventional bulk methods, this analysis identified two SNPs containing CREs: one linking *CTLA4* and *ICOS*, and the other linking *CD28, CTLA4*, and *ICOS* (Table S7)*.* This highlights the context-specific enhancer sharing among co-regulated immune checkpoint genes.

A study using extensive bulk functional and computational approaches recently implicated *BCL2L11* as a novel causal target of the RA-associated *ACOXL* locus on chromosome two.^43^ Our single-cell analysis independently supports this link, while also revealing additional associations and specificity to certain CD4^+^ T cell populations. Whilst we found one variant, rs10185010, was linked to *ACOXL* in unstimulated cells, *ACOXL* expression is limited to non-immune tissues and thus functionally questionable. Upon stimulation, the same SNP-containing peak links to *BCL2L11,* along with two additional peaks containing rs151158957 and rs13401811 (Figure 5A); the latter previously reported as causal. In our data, rs151158957 showed the strongest correlation, with peak accessibility highest in IL-10^+^ Th1-like cells, whilst the region harboring rs13401811 exhibited relatively uniform chromatin accessibility (Figure S7F). Notably, *BCL2L11* was also identified as an HRG, with 27 and 22 peak-to-gene linkages in eRA and rRA, respectively.

**Figure 5:**
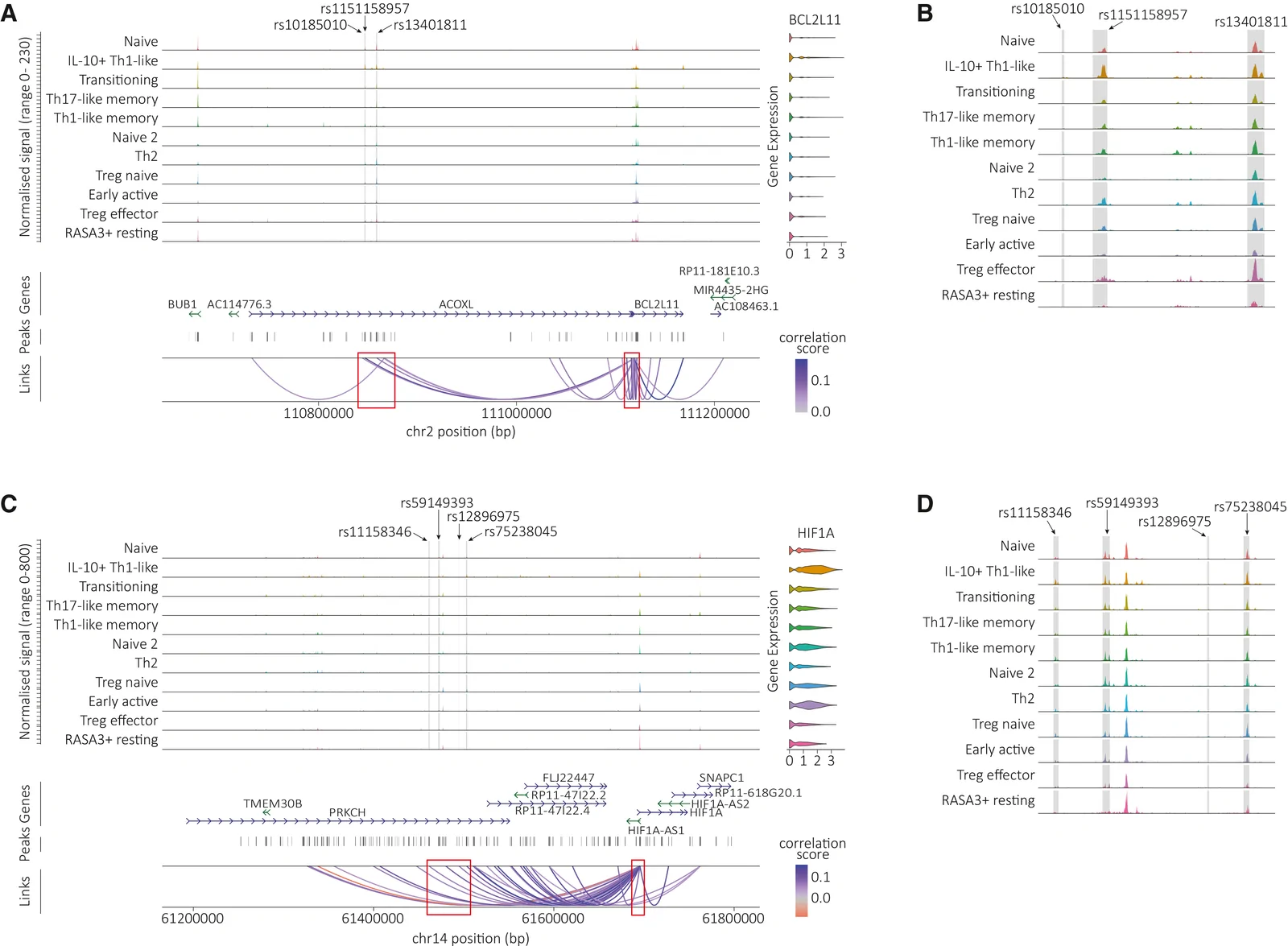
Functional annotation of RA risk variants (A) The chromatin accessibility landscape around the *ACOXL* susceptibility locus shown as cell type-specific pseudobulk ATAC-seq coverage tracks, generated using peak-to-gene linkage calculated using the full dataset. Below the coverage tracks, gene annotations and called ATAC-seq peaks are provided. Significant peak-to-gene linkages within this region are depicted as arcs connecting distal peaks to associated gene promoters. ATAC-seq peaks containing RA GWAS variants are highlighted in gray. To the right, *BCL2L11* expression levels are shown as violin plots across CD4^+^ T cell subsets. (B) ATAC-seq track from A, zoomed in on the regions containing the GWAS risk SNPs. (C) Same as in A but for the *PRKCH* susceptibility locus, generated using peak-to-gene linkages calculated within stimulated cells. To the right, *HIF1A* expression levels are shown as violin plots across CD4^+^ T cell subsets. (D) ATAC-seq track from C zoomed in on the regions containing the GWAS risk SNPs.

Beyond previously reported loci, the *PRKCH* region on chromosome 14 (Figure 5B) illustrates a potentially novel association. *PRKCH* expression is mainly limited to non-immune tissues, again making this annotation questionable. Here, we identify four CREs containing SNPs in LD with lead variant rs146492555. In eRA, only one peak (containing rs75238045) was linked to PRKCH, but following stimulation, this peak and three others, containing rs1158346, rs59149393, and rs12896975, were also linked to *HIF1A*, a gene with important immune regulatory roles. The strongest correlation was for rs75238045, while rs12896975 showed higher accessibility in naive Tregs (Figure S7G). *HIF1A* was also identified as an HRG, with 32 and 27 peak-to-gene linkages in eRA and rRA, respectively, supporting its potential as the effector gene at this locus.

Finally, to further investigate disease-stage differences in genetic regulation, we focused on CREs identified exclusively in eRA or rRA cells (Figure S7A). Most CREs were shared across analyses or detected in multiple modeling contexts (full dataset, unstimulated cells, stimulated cells, eRA, or rRA), suggesting broadly conserved regulatory architecture across disease stages. However, a small subset of CREs were uniquely detected within a single disease stage. In eRA-specific CREs, we observed 8 SNPs mapping to 7 peaks, whereas in rRA-specific CREs, we identified 12 SNPs mapping to 8 peaks, with most links assigning to a single gene (Table S7).

In rRA, disease-specific CREs were observed at loci involving TRAF1, PTGER4, RBM17, MAP4K2 and DGKH. The TRAF1 locus on chromosome 9, a well-established RA susceptibility region, exhibited multiple connections in our dataset, including several independent links to TRAF1 and multi-gene assignments involving TRAF1 and STOM. Importantly, two CRE interactions at this locus were detected exclusively in rRA: one CRE containing four SNPs linked to TRAF1 and another single-SNP CRE linked to CNTRL. In addition, an rRA-specific CRE containing two SNPs was identified at the MAP4K2 locus on chromosome 11, a key regulator of MAP kinase signaling in immune cells. This interaction was not detected in any other modeling context, indicating potential context-dependent resolution of a defined regulatory target within this pathway.

## Discussion

In this study, we present a systematic multi-step analytical workflow to characterize circulating CD4^+^ T-cells at a single-cell resolution, comparing rRA with the opposite end of the RA disease continuum, which could provide insight into refractory disease. By resolving cellular heterogeneity and mapping chromatin accessibility and gene expression at high resolution, we observe cell populations, transcriptional programs and regulatory landscapes that differ between the two disease stages, providing new insights into mechanisms which may have contributed to treatment resistance in rRA.

Although global regulatory patterns and overall cell proportions appear broadly similar between disease stages, a consistent theme emerges across multiple analyses, namely that naive CD4^+^ T cells show distinct and functionally relevant differences in rRA. This population, which is traditionally viewed as a relatively inert precursor pool, displays signs of heightened transcriptional priming, altered regulatory control, and dysregulated activation responses; all of which may contribute to immune dysregulation and chronic inflammation in rRA and associate with the challenges observed in treating this group of patients.

Despite limited differences in cell type proportions, gene expression profiling showed broader epigenetic and transcriptional changes in rRA, indicative of a more inflammatory phenotype. In rRA, the bulk of naive cells (larger naive population on UMAP) were enriched for cytoskeleton rearrangement programs, which may potentially raise the TCR activation threshold and negative regulation of cell fate decisions, suggesting an intrinsic attempt to restrain inappropriate lineage commitment and safeguard functional plasticity.^34^^,^^46^ Interestingly, the smaller subset of naive cells (Naive 2 on UMAP) in rRA exhibited histone demethylation as well as reduced responsiveness to stimulation; this suggests a chromatin-poised yet hyporesponsive stimulatory state, possibly as a means of preventing overactivation. In an inflammatory environment, these cells have the potential to either rapidly activate (if additional signals can overcome hyporesponsiveness) or drift toward exhaustion under chronic stimulation.

At the level of CREs and ranking linkage density to identify HRGs, irrespective of CD4^+^ population, eRA exhibited more peak-to-gene linkages to genes involved in activation and signaling (e.g., *CAMK2D*, *CBLB*, *ICOS*, *RBPJ*, *SAT1B*, *LRRN3*), suggesting tighter regulatory control in early disease. *SATB1* is particularly interesting due to its role as a genome organizer of CD4^+^ lineage commitment.^32^ When focusing on HRG expression at the population level, rRA showed fewer linkages and reduced expression of key regulatory genes on naive populations. This included *FOXP1*, which maintains quiescence and a naive phenotype^34^ and is crucial in maintaining the regulatory function of Tregs by regulating *FOXP3* expression.^47^ As well as *ICOS*, which supports Treg survival and function and is described to regulate IL-10–dependent suppression.^35^ Additionally, whilst under active regulation in eRA, we observed no links to *CCR2* in rRA, which could decrease cell chemotaxis to inflamed joints and has also been shown to regulate the effector/regulatory T lymphocyte ratios in mice.^33^^,^^48^ Collectively, these findings suggest altered regulatory architecture in rRA that may contribute to dysfunctional regulation of activation, amplify effector potential, and compromise immune resolution.

While global CRE-based regulatory modules were broadly conserved across disease stages, the genes they are regulating and the pathways enriched within these modules differed. These data show that the rRA group has greater pathway enrichment and dispersion across modules, suggestive of a more heterogeneous and active regulatory landscape. This landscape is dominated by genes that support sustained cytokine production and cellular persistence, which is consistent with chronic effector activity regulated by phosphatase-mediated feedback.^49^ These features may underlie the challenges associated with treating rRA where one pathway does not dominate. In addition, enrichment for ribosome biogenesis and translation corroborates the evidence that self-reactive T lymphocytes upscale their translational machinery and likely increase inflammation.^50^ Notably, *ZBTB7B* (ThPOK) was found to be enriched specifically in rRA (rRA module 6). *ZBTB7B* is a lineage-stabilizing *BTB-ZF* TF that preserves helper identity by repressing *BLIMP-1* and *RUNX3* (which lead to short-lived or exhausted effector states) and is pivotal for the emergence and function of memory CD4^+^ T-cells.^36^^,^^51^ Here, its exclusive up-regulation in refractory memory subsets suggests that these cells may be retaining a robust helper-memory phenotype, functional fitness, cytokine competence, and be resisting exhaustion checkpoints; thus potentially contributing toward the “difficult-to-treat” state.^52^

Functional responses to TCR stimulation further support these differences. Among the four populations with stage-specific transcriptional responses, only the larger naive population and Th1-like cells yielded sufficient numbers of differentially expressed genes for downstream analysis. Consistent with previous literature in the context of autoimmunity—including multiple sclerosis^53^ and systemic lupus erythematosus^54^—naive CD4^+^ T-cells in eRA showed robust down-regulation of genes controlling immune-synapse assembly, cytoskeleton remodeling and TCR signaling. In rRA, a blunted downregulation of the above-mentioned pathways suggests desensitization of these signaling cascades, which might contribute to dysfunctional, chronic activation. We corroborated our findings by identifying a putative hallmark of refractory naive cells in *BIRC2*, an anti-apoptotic E3 ligase whose knockdown mitigates inflammation and necroptosis in LPS-stimulated human chondrocytes C28/I2 cells^55^—hinting at altered survival signaling prior to stimulation in rRA by limiting immune cell turnover.

Motif enrichment of stimulation-responsive peaks revealed important TFs that may underlie the divergent responses of naive CD4^+^ T-cells between disease stages. In eRA, naive cells show strong enrichment for *STAT3, STAT1*, *FOS::JUN*, *CREB1*, and ATF3 motifs—TFs known to coordinate differentiation, activation, and immune regulation in response to inflammatory signals. Their reduced activity in rRA, suggests transcriptional exhaustion supporting that they may fuel chronic inflammation and therapy resistance. Modulation of these pathways may therefore represent an area for future investigation in treatment-refractory RA. Extending these findings, we further observed that TFs identified through motif enrichment were not only differentially enriched within stimulation-responsive regulatory regions but also showed disease stage-specific associations with downstream transcriptional response programs. Notably, STAT1 and STAT3 activity showed stronger negative associations with the stimulation-response program in eRA Naive CD4^+^ T-cells compared with rRA, suggesting altered/stronger coupling between STAT-associated regulatory accessibility and downstream transcriptional output across disease stages. Together, these results support a model of stage-dependent regulatory rewiring underlying divergent T cell responses to stimulation in RA. Interestingly, this analysis also highlighted loss of *FOXO4* enrichment in rRA Th1-like memory cells. *FOXO4* is known to restrain IFN-γ production and inhibit NF-κB nuclear import, thereby limiting effector functions.^56^^,^^57^ Inactivation of *FOXO4* in synovial tissues has been shown to negatively correlate with high levels of serum acute phase reactants,^58^ suggesting that loss of *FOXO4* may be removing a critical regulatory brake in rRA.

In addition to these findings, our analyses spotlighted genes with potential therapeutic relevance. Unique targeting by active TFs and higher expression of *LGALS3* was observed in rRA, particularly in memory-like and effector Tregs, consistent with its known role in supporting differentiated T cell functions.^59^ Interestingly, lower serum levels of *LGALS3* have been found to correlate with better physical function in RA^60^ and given that Galectin-3 inhibitors have shown promise in fibrotic and inflammatory models,^61^ these agents may therefore warrant further exploration in the context of drug repurposing in rRA. Additionally, integration of GWAS data identified *HIF1A* as a novel causal gene in RA. HIF-1α has been reported as a driver of RA pathology through its roles in metabolic reprogramming, inflammatory signaling and synovial tissue remodeling. Interestingly, elevated HIF-1α has been linked to the development of resistance to treatments in cancer studies, and clinical trials using HIF inhibitors have shown promising results, but can be associated with adverse side effects.^62^ Notably, a calcium phosphate-based anti-HIF1α siRNA has also demonstrated promising therapeutic effects in RA models.^63^ In this context, we also observed a small number of rRA-specific CREs at immune signaling loci, including TRAF1 and MAP4K2, suggesting that disease-stage-stratified regulatory mapping may further refine GWAS signal interpretation at selected loci and help prioritize potential therapeutic targets, although these observations are limited by sample size and should be interpreted cautiously.

This study offers several advantages. By applying single-cell multiomic profiling, we achieved high-resolution characterization of both the transcriptional state and chromatin landscape in CD4^+^ T cells from patients with eRA and rRA, providing granular insights into the mechanistic hypotheses underlying disease pathogenesis. A known challenge in transcriptomic profiling is the incomplete resolution of naive vs. central memory CD4^+^ T cells using canonical markers such as CCR7, CD27, CD28, IL7R, and SELL. In our dataset, these populations showed some separation at the RNA level, with CCR7 and CD27 showing the most consistent differences, while IL7R and CD28 were not informative and SELL showed limited separation at the transcript level. An advantage of using multi-modal RNA and ATAC-seq profiling is that chromatin accessibility-derived gene activity provided additional resolution where RNA-based signal was less discriminative, strengthening the delineation of naive-associated regulatory programs and supporting a continuum-based interpretation of naive-to-memory differentiation. This integrative approach also allowed us to link regulatory elements to gene expression and uncover functional CREs—notably, only 58.2% of CREs were linked to their nearest gene, highlighting the advantage of this approach over proximity-based methods. The inclusion of ATAC-seq data further enabled the integration of GWAS variants, refining the functional interpretation of genetic susceptibility loci within disease-relevant cell types. Importantly, this could be resolved within distinct populations that bulk approaches overlook, enabling direct identification of functional targets without the need for extended validation pipelines. For example, this study identified additional regulatory SNPs in the *ACOXL* region which were missed in bulk analyses.

To summarize, this study showed distinct regulatory and transcriptional features of CD4^+^ T cells in eRA and rRA, with naive CD4^+^ T cells consistently showing alterations which may be associated with treatment-refractory disease. Despite broadly similar global regulatory patterns across disease stages, rRA is marked by dysregulated gene expression, altered responses to stimulation, and rewired transcription factor activity, particularly within naive populations. These shifts are consistent with a transition from acute immune activation to a more persistent, chronic inflammatory state, which may be associated with treatment resistance. These results, to be replicated in larger studies, highlight transcriptional programmes and putative drug targets which may provide a basis for future therapeutic strategies aimed at restoring immune regulation in rRA.

### Limitations of the study

However, several limitations should also be noted. The peak-to-gene linkage method used is inherently gene-centric, relying on gene expression to anchor regulatory connections. Because expression data are collapsed into a single gene-level measure, it may overlook interactions specific to alternative isoforms. Additionally, this approach may overlook independent enhancer elements not directly linked to gene expression or accessible regions not linked to a gene and thereby potentially underestimating the complexity of regulatory landscapes. It should also be noted that we used the default ±500 kb window to link peaks to genes and so may have limited our ability to detect long-range peak-gene interactions and thus missed some distal regulatory effects. Sample size was also limited, restricting the generalisability of these findings and may have limited our ability to fully account for potential confounding by age and serostatus, despite the use of covariate-aware modeling and variance decomposition approaches; therefore, validation in larger independent cohorts is required. Analysis was confined to blood samples, which may exclude tissue-resident populations likely to play key roles in RA; however, blood remains the most clinically accessible sample type. Additionally, stimulation was performed under a single, controlled condition, which may have overlooked context-specific CREs that could nominate further risk variants. Broader profiling across multiple populations of cells and activation states may reveal additional disease-relevant regulatory elements. Finally, while patients were stringently classified into early or refractory disease groups, those in the latter group have received a variety of targeted therapies, which may have left an “immunological scar” and could contribute to the subtle regulatory and transcriptional changes we observed, thus complicating direct comparisons of disease-intrinsic states. However, differential expression analyses were performed using a pseudobulk framework and thus accounted for between-patient variability. Additionally, variance decomposition suggested only a modest contribution of treatment burden to transcriptional variability, although overlap with age and disease activity limited attribution of treatment-versus disease-intrinsic effects. Importantly, stimulation state and cell identity remained substantially larger drivers of transcriptional variation. Accordingly, the refractory-associated signatures should be considered exploratory and warrant validation in larger longitudinal cohorts.

## Resource availability

### Lead contact

Further information and requests for resources should be directed to and will be fulfilled by the lead contact, Samantha L Smith (samantha.smith-4@manchester.ac.uk).

### Materials availability

This study did not generate any new reagents or materials.

### Data and code availability

• Raw sequencing data have been deposited at NCBI’s Gene Expression Omnibus repository (GEO:GSE317456) and are publicly available as of the date of publication.
• This paper does not report original code.
• Any additional information required to reanalyze the data reported in this paper is available from the lead contact upon request.

## Acknowledgments

We are very grateful to all the patients who consented to participate in this study and to all the nurses who helped collect the samples. We would like to thank Andy Hayes and Claire Morrisroe at the University of Manchester Genomic Technologies Core Facility for their help with the single cell sequencing as well as Gareth Howell at the University of Manchester Flow Cytometry Core Facility. We also acknowledge assistance given by IT Services and the use of the Computational Shared Facility at the University of Manchester. Graphical abstract created in BioRender. Smith, S. (2026) https://BioRender.com/1qtz8qx.

This research was funded by the 10.13039/100014653NIHR Manchester Biomedical Research Centre (NIHR203308) and the 10.13039/100010269Wellcome Trust (207491/Z/17/Z) and supported by the CfGG Arthritis UK (grant no 21754) and BRC Musculoskeletal Theme small pump priming award. MHB is a 10.13039/501100000272National Institute for Health and Care Research (10.13039/501100000272NIHR) Senior Investigator and is supported by the 10.13039/100014653NIHR Manchester Biomedical Research Centre (NIHR203308). YT is part-funded by the 10.13039/501100010767‘3 TR (Taxonomy, Treatment, Targets, and Remission') Innovative Medicines Initiative 2 Joint Undertaking under grant agreement no 831434. The views expressed are those of the author(s) and not necessarily those of the NHS, the NIHR or the Department of Health and Social Care.

## Author contributions

Conceptualization: M.B., G.O., P.M., and S.E.

Funding acquisition: M.B., G.O., and P.M.

Data acquisition: R.S., Y.T., A.A., I.L., N.N., S.L.S., and S.R.

Analysis: S.L.S., S.R., P.M., J.B., K.P., and C.F.Y.

Writing - original draft: S.L.S. and S.R.

Writing - review and editing: All authors reviewed and edited the manuscript.

All authors read and approved the final manuscript.

## Declaration of interests

The authors declare no competing interests.

## STAR★Methods

### Key resources table

**Table:** 

REAGENT or RESOURCE	SOURCE	IDENTIFIER
**Antibodies**		

anti-CD3e (UCHT1)	Life Technologies	Q10054; AB_11180459
anti-CD4 (SK3)	BD Biosciences	562970; AB_2744424
anti-CD69 (FN50)	Biolegend	310906; AB_314841
Zombie UV viability dye	Biolegend	423107

**Chemicals, peptides and recombinant proteins**		

RPMI-1640	Sigma-Aldrich	R8758
Fetal Bovine Serum (FBS)	Thermo Fisher Scientific	A5256801
Penicillin-Streptomycin	Sigma	P4333
DynaBeads Human T-activator CD3/CD28	Thermo Fisher Scientific	11131D
CD4^+^ T cell isolation kit	Stemcell Technologies	17952
Fc Human TruStain FcX	Biolegend	422301
Phosphate Buffered Saline (PBS)	Sigma-Aldrich	D8537
Trypan Blue	Thermo Fisher Scientific	15250061
DTT	Sigma-Aldrich	646563
RNase Inhibitor	Sigma-Aldrich	3335399001
Nuclease free water	Invitrogen	AM9938
Tris-HCl	Sigma-Aldrich	T2194
NaCl	Invitrogen	AM9760G
MgCl_2_	Sigma-Aldrich	M1028
BSA	Sigma-Aldrich	A7030
Tween 20	Bio-Rad	1662404
Nonidet P40 Substitute	Sigma-Aldrich	74385
Digitonin	Thermo Fisher Scientific	BN2006

**Biological samples**		

Human peripheral blood mononuclear cells (PBMCs)	RA patients	N/A

**Critical commercial assays**		

Nuclei Buffer (20X)	10x Genomics	2000153/200
Chromium Next GEM kit and Single Cell Multiome ATAC + Gene Expression reagents bundle (v1.0)	10x Genomics	P/*N* 1000230, P/*N* 1000285
Illumina Infinium HumanCoreExome-24 BeadChip kit (v1.4)	Illumina	20039223

**Deposited data**		

Raw scRNA-seq data files	This Paper	GEO: GSE317456

**Software and algorithms**		

FlowJo v10.10.0	BD Biosciences	https://flowjo.com/flowjo10/download
GenomeStudio v2.0	Illumina	N/A
PLINK v1.9	Christopher C Chang et al., 2015	https://www.cog-genomics.org/plink/1.9/
Cellranger v2.0.1	10x Genomics	https://www.10xgenomics.com/support/software/cell-ranger/downloads
popscle suite v0.1-beta	N/A	https://github.com/statgen/popscle
R v4.2.2	R Development Core Team	https://www.r-project.org/
Seurat v4.3	Hao et al.^64^	https://satijalab.org/seurat/articles/install_v5
Signac v1.10	Stuart et al.^65^	https://stuartlab.org/signac/articles/install
motifmatchr package v1.2	Schep 2025	https://bioconductor.org/packages/release/bioc/html/motifmatchr.html
DESeq2 package v1.38.3	Love et al.^66^	https://bioconductor.org/packages/release/bioc/html/DESeq2.html
clusterProfiler package (v4.6.2)	Wu et al.^67^	https://bioconductor.org/packages/release/bioc/html/clusterProfiler.html
TopGo (v2.5)	N/A	https://bioconductor.org/packages/2.5/bioc/html/topGO.html
GraphPad Prism v9.3.1 and v10.4.2	GraphPad Software	https://www.graphpad.com
Biorender	N/A	https://www.biorender.com/

**Other**		

BD FACSymphony A5 flow cytometer	BD Biosciences	N/A
Illumina iScan System	Illumina	N/A

### Experimental model and study participant details

#### Study design

This study was conducted at the University of Manchester and Kellgren Center for Rheumatology, Manchester Royal Infirmary as part of the Precision medicine across the disease continuum to prevent and treat Rheumatoid Arthritis (PRECISE-RA) program (REC 20/SS/0090; NHS Lothian) and sponsored by the University of Manchester. PRECISE-RA is an observational cohort and experimental study in people with RA, recruiting patients at any point along their disease continuum. Written informed consent was obtained from all participants. All conduct was carried out in-line with the Declaration of Helsinki principles.

#### Patients

Six patients with RA, all fulfilling the 2010 ACR/EULAR classification criteria, were recruited: three treatment-naïve eRA (≤12 months symptom duration) and three rRA patients (defined as per EULAR definition of difficult-to-treat RA,^68^ i.e., inefficacy of at least 2 different mechanism of action biologic/targeted synthetic DMARDs and signs of active disease). All 6 participants were seropositive and had active disease as defined as disease activity score of 28 joints (DAS28)-erythrocyte sedimentation rate (ESR) > 3.2.

### Method details

#### Sample processing and flow cytometry

Peripheral blood mononuclear cells (PBMCs) were extracted from whole blood, collected into EDTA tubes, using ficoll density gradient centrifugation and cryopreserved for later use. Primary CD4^+^ T-cells were negatively selected using the CD4^+^ T cell isolation kit (Miltenyi Biotec). Cells were seeded (1e10^6^ cells/ml) in media (Gibco RPMI 1640 supplemented with 10% FBS and 1% penicillin/streptomycin). Subsequently, half were stimulated with Dynabeads Human T-Activator CD3/CD28 (ThermoFisher Scientific) for 4-h as per the manufacturer’s protocol, while the other half remained unstimulated. Following stimulation, beads were removed by magnet separation.

To assess purity and cellular activation (Additional file 2: Figures S1A and S1B), cells were stained with Zombie UV viability dye (BioLegend) for 20 min at 4°C. Cells were blocked with Fc Human TruStain FcX (BioLegend) for 10 min at 4°C and then stained with Qdot605-labelled anti-CD3e (clone UHTC1; Life Technologies), BV510-labelled anti-CD4 (clone SK3; BD Biosciences), and PE-labelled anti-CD69 (clone FN50; BioLegend). Finally, samples were washed, fixed and assessed by the high-parameter BD FACSymphony A5 flow cytometer, analyzed with the FlowJo software v10.10.0 (BD Life Sciences). Statistical significance was determined using a two-tailed paired Student’s t test using GraphPad Prism v10.4.2 (GraphPad Software, Boston, Massachusetts USA). Asterisks were used to represent *p*-values: ∗∗∗∗*p* < 0.0001. A *p*-value of 0.05 was considered significant.

#### Nuclei isolation, library construction and sequencing

Per stimulation condition, CD4^+^ T-cells from six individuals were pooled in equal proportions. Nuclei were isolated following the 10x Genomics Nuclei Isolation Protocol (CG000365, Rev B) and resuspended to achieve a target recovery of 10,000 nuclei per condition. Libraries were prepared using the Chromium Next GEM Single Cell Epi Multiome ATAC + Gene Expression User Guide (10x Genomics, Pleasanton, CA), according to the manufacturer’s instructions. Library quality was assessed using a D1000 TapeStation system (Agilent). Final libraries were sequenced on an Illumina NovaSeq 6000 system (Novogene, UK). Single-nucleus ATAC-seq (snATAC-seq) libraries were sequenced on one SP flow cell, and single-nucleus RNA-seq (snRNA-seq) libraries were sequenced on one lane of an S4 flow cell following manufacturers’ recommendations.

### Quantification and statistical analysis

#### Genotyping and demultiplexing using demuxlet

Genotyping was performed using the Illumina Infinium HumanCoreExome-24 BeadChip kit (Illumina, San Diego, CA, USA), according to the manufacturer’s guidelines. Genotype calling was carried out using GenomeStudio software (Illumina, San Diego, CA, USA). Standard QC^69^ was conducted using PLINK v1.9 using the following exclusion criteria: sample and SNP call rate <0.98, SNPs with minor allele frequency (MAF) < 0.01, discrepancy between genetically inferred sex and clinical recorded sex and non-autosomal SNPs. Imputation was carried out using the HRC v1.1 reference panel (GRCh37/hg19) on the Michigan Imputation Server.^70^ Following imputation, SNPs with MAF<0.01 and an imputation r2 score lower than 0.5 were removed.

Raw FASTQ files were processed using Cell Ranger ARC (v2.0.1),^71^ using default settings to map reads against the Human GRCh38 (GENCODE v32/Ensembl98) reference (2020-A, https://cf.10xgenomics.com/supp/cell-arc/refdata-cellranger-arc-GRCh38-2020-A-2.0.0.tar.gz), perform filtering, counting, peak calling, and produce a single cell feature count matrix for subsequent analysis.

Cell containing droplets called by Cell Ranger ARC were computationally demultiplexed using the dsc-pileup and demuxlet commands of the popscle suite (v0.1-beta) for ATAC-seq and RNA-seq libraries separately, using the imputed genotypes and filtered Haplotype Reference Consortium (HRC) panel (r1-1, MAF ≥0.01).

#### Quality control

In-depth quality control, pre-processing and analyses were conducted using Seurat (v4.3)^64^ and Signac (v1.10)^65^ in R (v4.2.2). Quality control and pre-processing were performed separately for each stimulation condition.

Ambient droplets were excluded based on demuxlet droplet classification.^72^ Discordant assignments between the RNA and ATAC assays were removed to ensure assay consistency. Additionally, predicted doublets identified by demuxlet were also excluded. Cells with low-quality ATAC or RNA profiles were removed based on the following criteria: high nucleosome signal (≥1.5), low and high total ATAC counts (≤1,500 or ≥60,000), low and high blacklist ratio (≤0.0005 or ≥0.05), low fraction of reads in peaks (≤51%), low TSS fraction (≤38%) and low and high total RNA counts (≤2000 or ≥15,000). The TSS enrichment threshold was higher for stimulated cells (≥14) compared to unstimulated cells (≥12). Cells with high mitochondrial RNA content (≥17% for unstimulated, ≥15% for stimulated) were also removed. We additionally applied Seurat’s reference mapping workflow, whereby we projected our data onto the publicly available multimodal reference dataset of 162,000 PBMCs.^64^ Cells predicted as non-CD4^+^ T-cells based on the transferred labels were excluded from downstream analyses. This left 7,997 unstimulated and 6,628 stimulated cells for analysis.

#### Pre-processing and integration

For the RNA assays, normalisation, variance stabilisation, scaling and variable feature selection were performed using Seurat’s SCTransform() function. Dimensionality reduction was performed RunPCA(). For the ATAC assays, peaks were re-called using MACS2 and normalised (RunTFIDF()), followed by feature selection (FindTopFeatures()) and dimensionality reduction using singular value decomposition (RunSVD()). Both modalities were clustered using Seurat’s graph-based unsupervised clustering framework and visualised using Uniform Manifold Approximation and Projection (UMAP) plots (RunUMAP(), FindNeighbors() and FindClusters()).

Following unsupervised clustering, batch effects associated with donor identity were observed in both modalities and corrected via Seurat’s integration pipeline which utilises SelectIntegrationFeatures(), FindIntegrationAnchors() and IntegrateData() functions. Following this, the two stimulatory conditions were integrated into a single Seurat object for joint clustering and downstream analysis.

Briefly, for the RNA integration, SCTransform() was performed, regressing out mitochondrial content and cell cycle genes. A total of 3,000 features were selected for integration and integration anchors were identified using all unstimulated donor samples as a reference. Integration was performed using 30 principal components. Dimensionality reduction and clustering was performed using 1:20 principal components with a resolution of 0.4.

For ATAC integration, peaks common to both conditions were taken forward for further analysis. Integration anchors were identified using all unstimulated donor samples as a reference and data integration was performed using 2:30 Latent Semantic Indexing coordinates. Clustering was performed using the LSI embeddings and a resolution of 0.5.

Finally, Weighted Nearest Neighbor analysis^64^ was performed to jointly integrate the modalities. For each cell, multimodal neighbors were calculated using FindMultiModalNeighbors() and the same dimensions as used above (RNA 1:20 and ATAC 2:30), followed by dimensionality reduction and clustering using a final resolution of 0.3.

#### Data analysis

All analyses were conducted using R (v4.2.2).

#### CD4^+^ T cell population annotation

Markers associated with each CD4^+^ T cell population were identified using Seurat’s *FindAllMarkers()* function, using a negative binomial test while controlling for donor sex as a latent variable. Gene activity scores were calculated using chromatin accessibility data using Signac’s *GeneActivity()* function. Transcription factor motifs were incorporated using the *AddMotifs()* function with the JASPAR2020 position frequency matrix (PFM) database.^73^ Differential TF motif activity analysis was performed based on c*hromVAR*^74^ deviation z-scores. Differential abundance analysis was performed using *scProportionTest* (v0.9). To assess the relative contribution of biological, technical and patient-level clinical variables to transcriptional heterogeneity, variance decomposition analysis was performed using the scater R package.^75^

#### Linkage of gene-regulatory elements to gene expression

Cis-Regulatory Elements (CREs) were linked to potential gene targets using Signac’s *LinkPeaks()* function which employs a correlation-based approach between peaks accessibility and gene expression.^65^ For some analyses, only high-confidence peak-to-gene linkages were retained by excluding links with a Pearson correlation coefficient <0.1. For GWAS annotation, a consensus peak-to-gene link set was created by combining all identified peak-to-gene linkages and removal of duplicate links, resulting in a consensus set of 26,345 linkages.

#### Analysis of HRGs and CREs

Similar to Ober-Reynolds et al.,^37^ all expressed genes were ranked according to the number of peak-to-gene linkages. A cut-off near the inflection point (‘elbow on the plot’) of 20 linked peaks-per-gene was used to define a highly regulated gene (HRG). We also explored co-accessible regulatory hubs. Firstly, peak-to-gene linkages were filtered based on differential chromatin accessibility between populations. The remaining peaks were then clustered (*k*-means clustering) to identify co-accessible hubs/modules. We visualised the gene expression of linked genes and performed Gene Ontology (GO) enrichment analysis on the top 250 CREs per *k*-means cluster. We use Signac’s *FindMotif()* function to measure the enrichment of TF motifs within *k*-means clusters.

#### Differential TF activities and their target genes

To reduce noise inherent in sparse single-cell data and enable robust correlation, cells were aggregated into pseudobulked samples. Using a *k-*nearest neighbor (*k* = 50) graph derived from Latent Semantic Indexing (LSI) embeddings, approximately 500 low-overlapping pseudobulk aggregates were constructed. For each pseudobulk sample, the mean normalised chromatin accessibility signal was calculated and the same cell groupings were subsequently applied to gene expression and *chromVAR* deviation scores for integrative analysis. Pseudobulked samples were used to calculate the Pearson correlation coefficient between candidate TF motif activities and the gene expression of all expressed genes. Population-specific activity of TFs was assessed by calculating the maximum difference in *chromVAR* deviation z-scores across CD4^+^ T cell populations for each motif. For candidate TFs, a linkage score (LS) was computed to prioritise potential target genes, following the approach described by Ober-Reynolds et al.^37^ Specifically, for every TF-gene pair, all peak-to-gene linkages were identified where the linked peak contained a matching TF motif. The LS was calculated as the sum of the squared peak-to-gene correlation coefficients multiplied by the motif match scores for each relevant peak. Motif scores were derived using the *motifmatchr* package (v1.2), which uses a log-odds scoring method. As a result, genes with multiple motif-containing linked peaks, stronger correlations, and/or higher-confidence motif matches received elevated scores. Additionally, a hypergeometric test was performed to assess motif enrichment across all linked peaks for each gene. Putative TF target genes were defined as those exhibiting both a TF motif–gene expression correlation >0.25 and an LS exceeding the 75th percentile across all genes. GO enrichment was then conducted using these target genes.

#### Differential expression analyses

Differential gene expression analysis was performed using the DESeq2 package (v1.38.3).^66^ A generalised linear model framework was applied to identify genes exhibiting expression changes associated with stimulation status (unstimulated or stimulated), disease stage (eRA or rRA), and their interaction (whilst accounting for sex as a covariate). Significance resulting from these comparisons were corrected for multiple hypothesis testing using the Benjamini-Hochberg false discovery rate (FDR) method (adjusted *p*-value < 0.05). The relevant contrasts were extracted as needed.

#### Differential stimulation-responsiveness

For the identification of stimulation-responsive genes differing by disease stage, we extracted the relevant contrast from the DESeq2 model explained above and GO enrichment was performed on genes exhibiting differential responses to stimulation (adjusted *p*-value < 0.05). To identify stimulation-responsive regulatory elements potentially driving these differences, by disease stage, peak-to-gene linkages were integrated with differentially accessible (DA) chromatin regions (identified using *FindMarkers()* function in Seurat, utilising a Wilcoxon rank-sum test). Peaks with a *p*-value < 1e−5 were considered DA. Peak-to-gene linkages were further filtered to retain only those with a linkage *p*-value <0.001. Stimulation-responsive linked DA peaks were annotated based on genomic location relative to gene features using the *TxDb* annotation package (v3.16). TF motif enrichment within stimulation-responsive linked peaks were determined using *FindMotifs()* and motifs with an adjusted *p*-value < 0.05 were considered significantly enriched.

To further investigate the relationship between enriched TF motifs and downstream transcriptional responses, chromVAR deviation scores were calculated for motifs enriched within stimulation-responsive peaks. A single-cell stimulation-response gene program score was generated using Seurat’s *AddModuleScore()* function, based on genes identified as significant in the DESeq2 interaction model, representing genes whose transcriptional response to stimulation differed between eRA and rRA. The score therefore represents a continuous axis summarising how strongly individual cells express this interaction-defined response signature relative to matched background genes. Correlations between chromVAR-derived TF activity and the stimulation-response gene program were computed separately within each disease group using Spearman correlation. Positive correlations indicate concordant relationships between TF activity and gene program scores across cells, whereas negative correlations indicate inverse relationships. In comparisons between disease groups, TFs close to the diagonal (y = x) showed similar TF-program relationships, whereas deviation from the diagonal indicated disease stage-specific differences in TF-program coupling. TFs exceeding a 1.0 standard deviation threshold from the diagonal were highlighted as showing the strongest differential associations.

#### GWAS annotation and prioritization of candidate SNPs

Lead SNPs from the 2022 multi-ancestry RA GWAS^42^ and SNPs in high linkage disequilibrium (LD; R^2^ > 0.8) were identified using plink v1.90b3.39 based on the Phase 3 1000 Genomes reference panel and restricting to individuals of European ancestry^43^; yielding a total of 2,818 variants. To link variants to target genes and prioritise candidate causal variants, peak-to-gene linkages were used to assign nearby genes to single nucleotide polymorphism (SNP)-containing peaks. Using this approach, 87 GWAS variants were identified with potential regulatory roles in CD4^+^ T-cells and linked to gene targets for further prioritisation. To visualise and interpret the regulatory context of RA GWAS variants, genomic regions of interest were plotted using Signac’s *CoveragePlot()* function. Peaks containing GWAS SNPs were highlighted by shading in gray. Permutation-based enrichment of CRE-SNP overlap was assessed using the *GWASPeakZtest()* function as implemented in Jiang et al., 2024, which compares observed SNP-peak overlaps to GC- and accessibility-matched background peak sets generated using chromVAR-based sampling.^76^ Partitioned SNP heritability was estimated using stratified LD score regression (LDSC v3.0.1).^77^^,^^78^ Summary statistics for were obtained from the GWAS Catalog (accession ID GCST90132223). Custom annotations corresponding to CREs identified in unstimulated and stimulated CD4^+^ T-cells were constructed. Partitioned heritability was estimated jointly with the baselineLD v2.2 model, which includes a comprehensive set of functional genomic annotations.

#### Visualisation and pathway enrichment analysis

Heatmaps were visualised using Seurat’s *DoHeatmap()* function, *ComplexHeatmap* (v2.14) or *ggplot2* (v3.4.1), depending on the data structure and visualisation requirements. In some instances, heatmap rows were clustered using *k-*means partitioning. Overlaps between sets of genomic features (e.g., genes, TF motifs) were visualised using plots generated with the *UpSetR* (v1.4) package. Biological pathway analysis was performed using *enrichGO()* from the *clusterProfiler* package (v4.6.2)^67^ or *TopGo* (v2.5), with all expressed genes used as the background set for enrichment testing. Some figure panels were created using GraphPad Prism Version 9.3.1 (GraphPad Software, Boston, Massachusetts USA).

## Supplemental information

Document S1. Figures S1–S7

Table S1. Top markers expressed per CD4^+^ T cell population, related to Figure 1

Table S2. List of genes differentially expressed by disease stage within each CD4^+^ T cell populations, related to Figure 1

Table S3. List of highly regulated genes and the number of peak-to-gene linkages per gene, calculated using: 1) full dataset, 2) eRA cells only and 3) rRA cells only, related to Figure 2

Table S4. List of putative target genes for eRA and rRA by TF, related to Figure 3

Table S5. List of drugs targeting target genes of interest, related to Figure 3

Table S6. By CD4^+^ T cell population, lists of genes differentially responding to stimulation between disease stages, related to Figure 4

Table S7. Annotation of CRE peaks containing RA GWAS SNPs, related to Figure 5
