# Supplementary material for: Exploratory single-cell multiomics suggests naive CD4+ T cell dysregulation in treatment refractory rheumatoid arthritis
Source: iScience. 2026 Oct 27;29(11):117949. doi: 10.1016/j.isci.2026.117949 (PMC13734958; doi:10.1016/j.isci.2026.117949)
Supplement: Supplementary file 1 [file mmc1.pdf]

iScience, Volume *29*

Supplemental information

Exploratory single-cell multiomics suggests naive CD4$^+$ T cell dysregulation in treatment refractory rheumatoid arthritis

Samantha L. Smith, Stefano Rossi, Rudresh Shukla, Chuan Fu Yap, Nisha Nair, Jake Butler, Yvonne Tan, Amirah Binti Mohammad Ariff, Ilinca Lazar, Khadijah Patel, Stephen Eyre, Maya H. Buch, Paul Martin, and Gisela Orozco

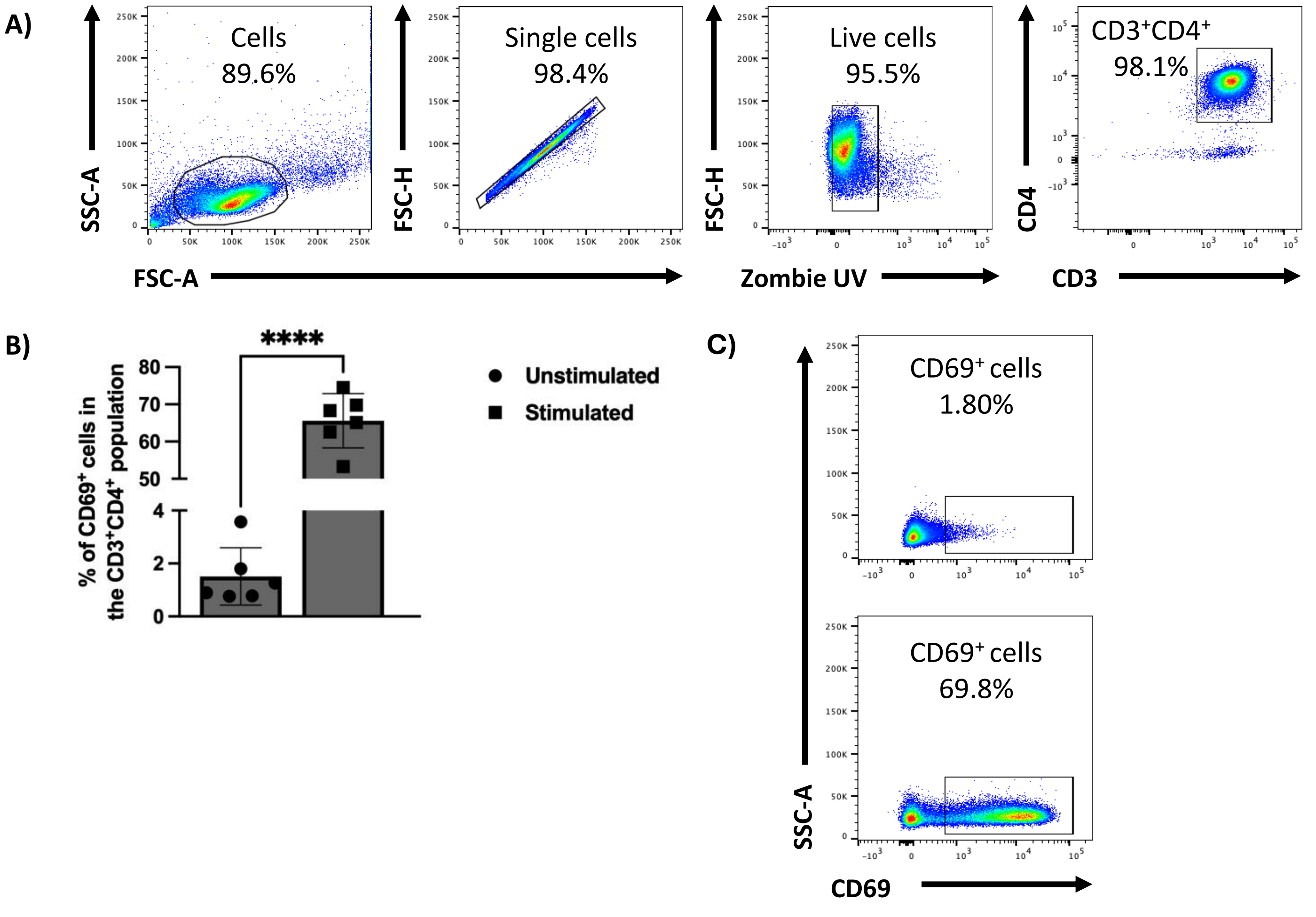

Fig. S1: Flow cytometry plots of purity and cellular activation

A) Representative flow-cytometric profile showing the CD4$^+$ gate within the magnetically bead-enriched CD4$^+$ fraction to verify enrichment efficiency. B) Bar graph showing the percentage of CD4$^+$ T-cells that up-regulate the early activation marker CD69 upon anti-CD3/CD28 beads *in-vitro* stimulation. C) Representative flow plot displaying the right-ward shift in CD69 fluorescence intensity in stimulated CD4$^+$ T-cells relative to their unstimulated counterparts. For stimulation quantification, significance was calculated using a two-tailed paired students t-test (****p<0.0001).

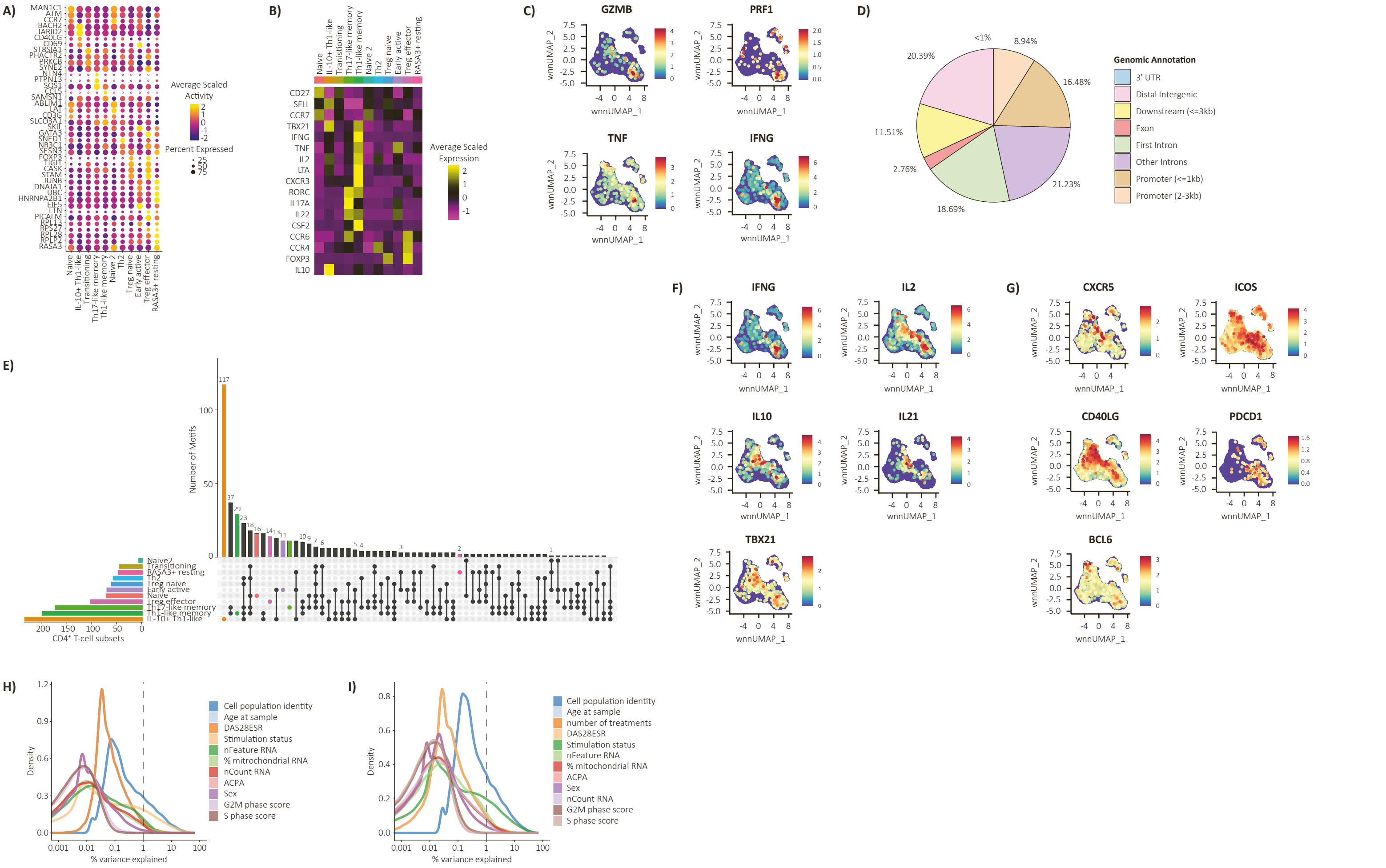

Fig. S2: Extended population annotation and TF motif analysis

A) snATAC gene activity scores for the markers shown in Fig. 1C. The colour encodes the average scaled gene activity values, ranging from low activity (purple) to high activity (yellow). B) Heatmap of gene expression for additional markers of interest used to annotate CD4⁺ T-cell populations. Populations are plotted in columns, and genes are shown in rows. Gene expression is colour coded using average scaled expression values per population, based on a z-score distribution, ranging from low expression (purple) to high expression (yellow). C) Featureplots showing the gene expression of selected markers used to phenotype TEMRA cells. Cells are ordered such that those with higher expression levels are plotted on top to improve visualization of positively expressing populations. Gene expression is color-coded, with higher expression levels indicated by warmer colours. D) Piechart showing the genomic annotation of called ATAC-seq peaks based on their proximity to gene features. Annotation was performed using the TxDb.Hsapiens.UCSC.hg38.knownGene database (genome build hg38). E) UpsetPlot showing the overlap in TF motif enrichment across CD4⁺ T-cell populations. The horizontal bars represent the total number of motifs enriched in each population (adjusted p-value<0.05), while the vertical bars indicate the number of motifs shared between subsets as defined by the connected dots below. Selected CD4⁺ T-cell populations are colour-coded corresponding to subset-specific motifs. The number of intersections was capped at 60 for visualisation. F) same as C) but showing the gene expression of selected markers to phenotype the predomominantly stimulated IL10+ Th1-like cell cluster. G) same as C) but showing the gene expression of selected markers to phenotype circulating T follicular helper (Tfh) cells. H) Variance decomposition analysis of CD4⁺ T-cell transcriptomes performed using the scater R package, illustrating the relative contribution of biological, technical and patient-level covariates to gene expression variability across all cells. I) same as H) but for refractory cases only and adding in the number of treatments recieved.

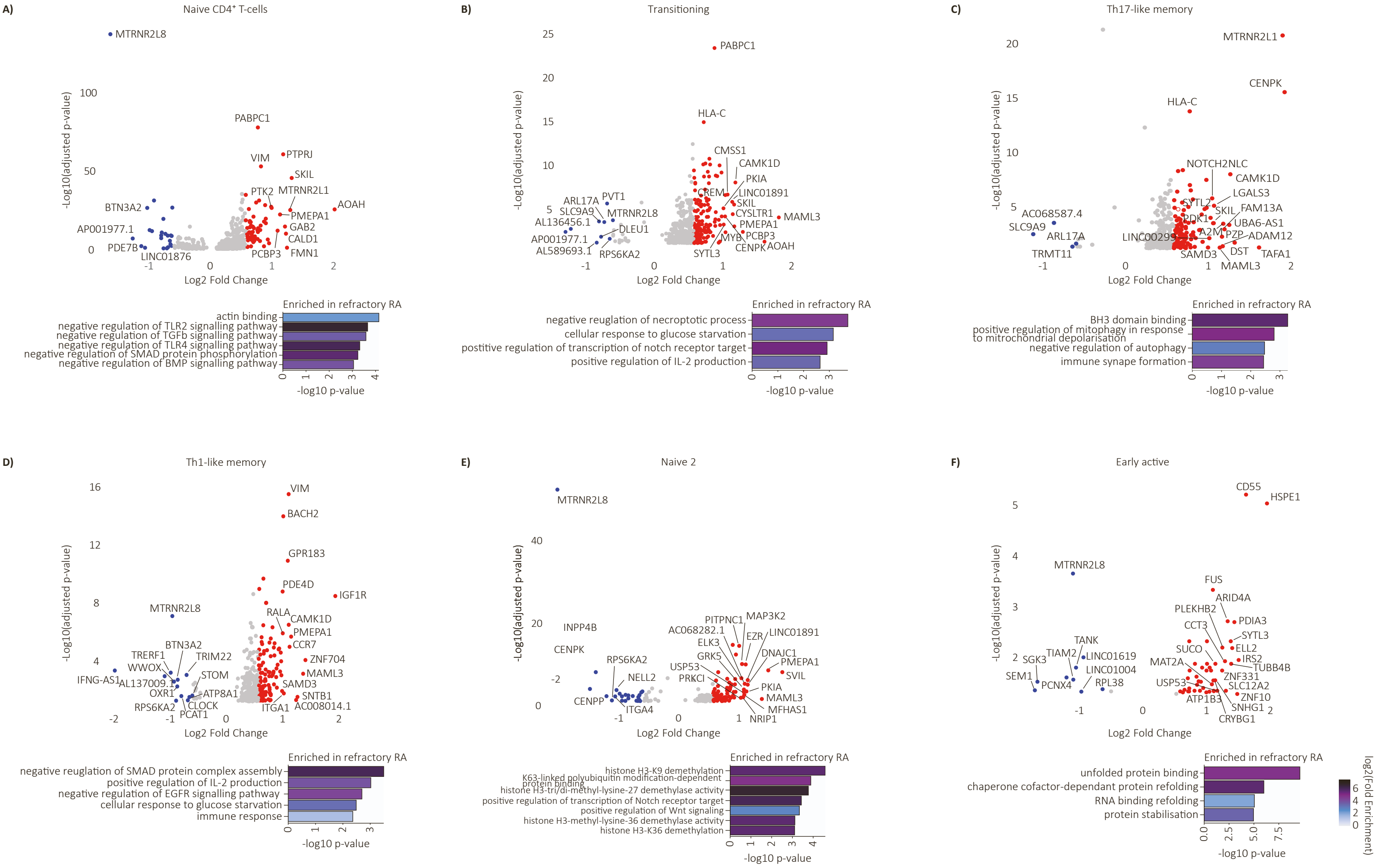

Fig. S3: Differential expression between disease stages

A) Volcano plots showing differential gene expression in naive CD4⁺ T-cells between early and refractory RA. Genes with adjusted p-values<0.05 and log2 fold-change>0.585 are coloured, with upregulated genes in refractory RA coloured red and upregulated genes in early RA coloured blue (grey represents non-significant genes). GO enrichment was performed using significant genes in refractory RA only (those in red). B) same as in A)but for transitioning CD4⁺ T-cells. C) same as in A) but for Th17-like memory CD4+ T-cells. D) same as in A) but for Th1-like memory CD4⁺ T-cells. E) same as in A) but for Naïve 2 CD4⁺ T-cells. F) same as in A) but for early active CD4⁺ T-cells.

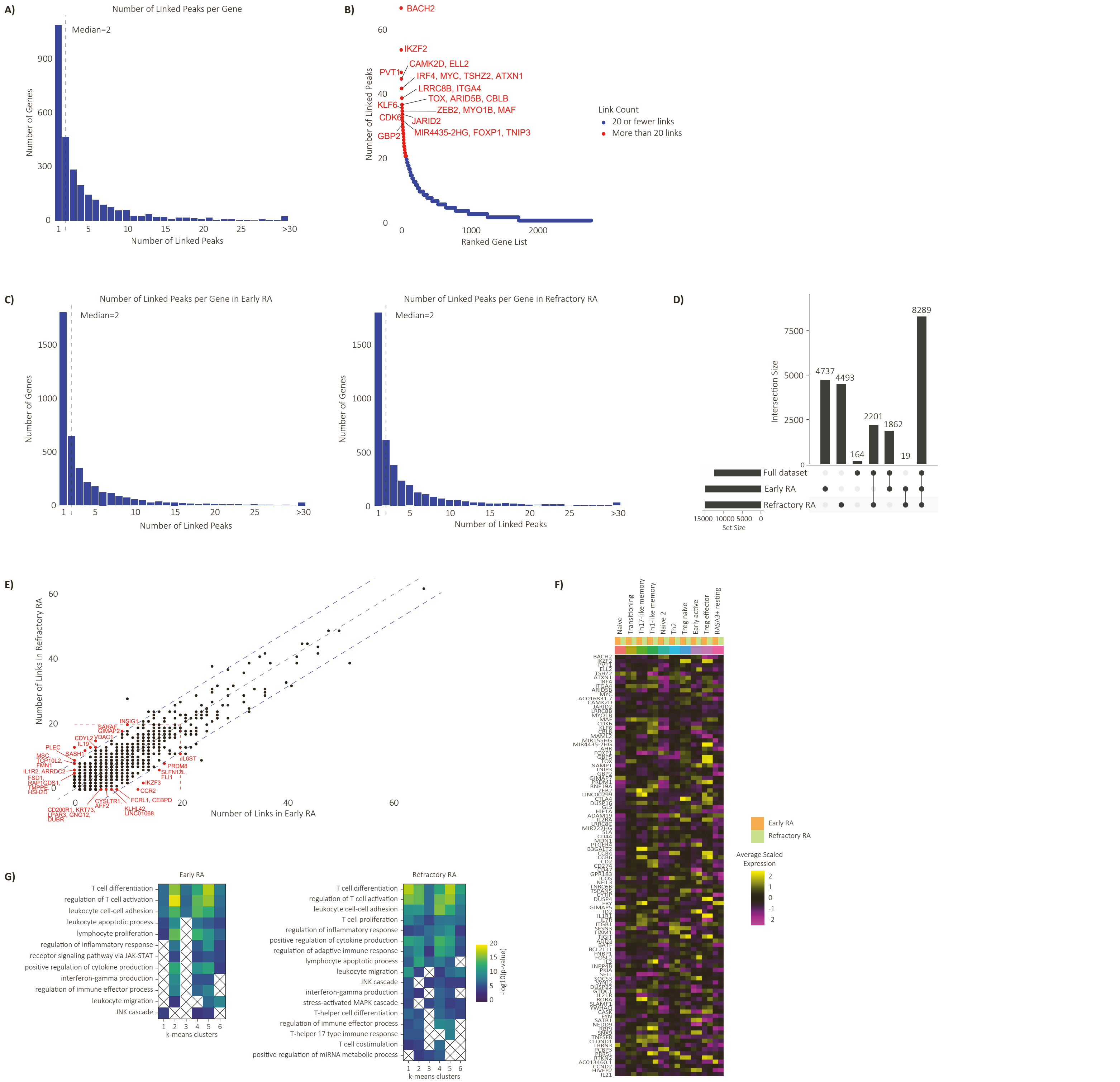

Fig. S4: Extended HRG and CRE analysis

A) Bar graphs showing distribution of the number of linked ATAC-seq peaks per gene across all cells. The x-axis is capped at 30, with genes having more than 30 linked peaks grouped into a single category (">30"). The blue bars represent the number of genes with a given number of linked peaks. A dashed vertical line indicates the median number of linked peaks per gene. B) Genes ranked by the number of peak-to-gene linkages identified for each gene using peak-to-gene linkages calculated using all cells: 78 HRGs had >20 peak-to-gene linkages. C) same as A) but for early RA (left) and refractory RA (right). D) UpsetPlot showing the overlap in peak-to-gene linkages between datasets. The horizontal bars represent the total number identified within each dataset), while the vertical bars indicate the number of unique or shared links between datasets as defined by the connected dots. E) Scatterplot showing the comparison of CREs identified in early RA (x-axis) and refractory RA (y-axis), focussing on less regulated genes (<20 peak-to-gene linkages). The axes show the number of peak-to-green linkages identified for each gene in early RA (x-axis) and refractory RA (y-axis). Significantly different CREs were based on residuals from the line of identity and defined as points exceeding ±1.5 standard deviations of the residuals or those displaying 0 links in one disease stage and >4 links in the other. F) Heatmap of gene expression for HRGs. Populations, split by disease stage (early RA in orange, refractory RA in green) are plotted in columns, and genes are shown in rows. Gene expression is colour coded using average scaled expression values per population and per disease stage, based on a z-score distribution, ranging from low expression (purple) to high expression (yellow). G) GO term enrichment for terms shared across k-means clusters (Fig. 2D). k-means clusters represent columns and rows represent GO terms. Early RA shown on the left and refractory RA shown on the right.

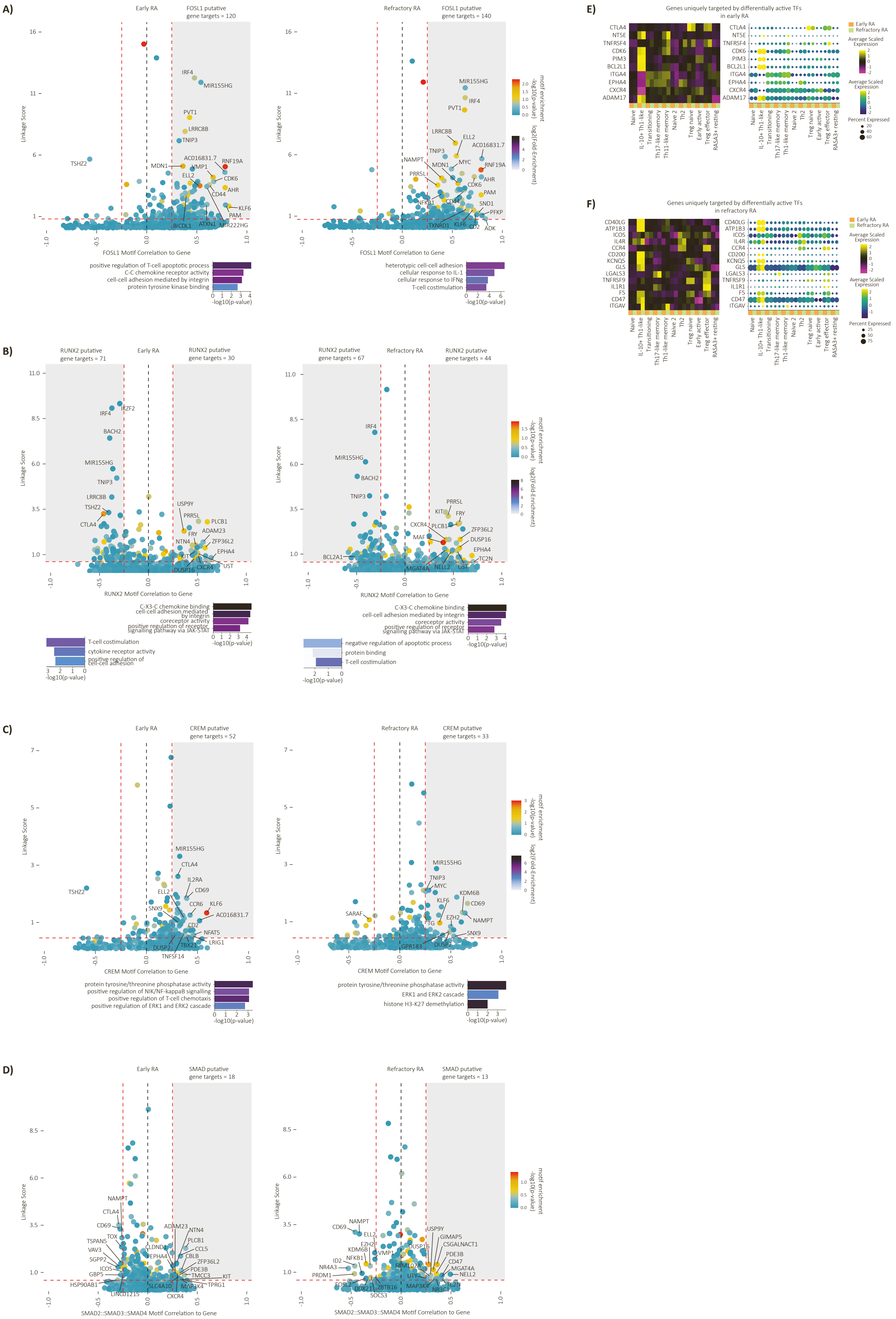

A)
Early RA
FOSL1 putative gene targets = 120
Refractory RA
FOSL1 putative gene targets = 140
Linkage Score
FOSL1 Motif Correlation to Gene
positive regulation of T-cell apoptotic process
C-C chemokine receptor activity
cell-cell adhesion mediated by integrin
protein tyrosine kinase binding
heterotypic cell-cell adhesion
cellular response to IL-1
cellular response to IFNg
T-cell costimulation
-log10(p-value)
motif enrichment -log10(p-value)
log2(Fold-Enrichment)
B)
RUNX2 putative gene targets = 71
Early RA
RUNX2 putative gene targets = 30
RUNX2 putative gene targets = 67
Refractory RA
RUNX2 putative gene targets = 44
RUNX2 Motif Correlation to Gene
C-X3-C chemokine binding
cell-cell adhesion mediated by integrin
coreceptor activity
positive regulation of receptor signalling pathway via JAK-STAT
T-cell costimulation
cytokine receptor activity
positive regulation of cell-cell adhesion
negative regulation of apoptotic process
protein binding
C)
Early RA
CREM putative gene targets = 52
Refractory RA
CREM putative gene targets = 33
CREM Motif Correlation to Gene
protein tyrosine/threonine phosphatase activity
positive regulation of NIK/NF-kappaB signalling
positive regulation of T-cell chemotaxis
positive regulation of ERK1 and ERK2 cascade
ERK1 and ERK2 cascade
histone H3-K27 demethylation
D)
Early RA
SMAD putative gene targets = 18
Refractory RA
SMAD putative gene targets = 13
SMAD2::SMAD3::SMAD4 Motif Correlation to Gene
E)
Genes uniquely targeted by differentially active TFs in early RA
CTLA4
NT5E
TNFRSF4
CDK6
PIM3
BCL2L1
ITGA4
EPHA4
CXCR4
ADAM17
Early RA
Refractory RA
Average Scaled Expression
Percent Expressed
Naive
IL-10+ Th1-like
Transitioning
Th17-like memory
Th1-like memory
Naive 2
Th2
Treg naive
Early active
Treg effector
RASA3+ resting
F)
Genes uniquely targeted by differentially active TFs in refractory RA
CD40LG
ATP1B3
ICOS
IL4R
CCR4
CD200
KCNQ5
GLS
LGALS3
TNFRSF9
IL1R1
F5
CD47
ITGAV

Fig. S5: Additional TF target genes and gene expression heatmaps for genes of interest

A) Identification of putative gene targets for FOSL1 in early RA (left) and refractory RA (right). The x-axis shows the Pearson correlation between the TF motif activity and gene expression for all expressed genes across CD4$^+$ T-cell populations. The y-axis shows the TF Linkage Score (for all linked peaks, sum of motif score scaled by linkage correlation). Colour of spots indicates the hypergeometric enrichment of the TF motif in all linked peaks for each gene. Putative gene targets are indicated in the shaded area (motif correlation to gene expression>0.25, linkage score>75th percentile). GO term enrichment for the gene targets are shown. B) same as A) but for RUNX2. C) same as A) but for CREM. D) same as A) but for SMAD2::SMAD3:SMAD4. E) Heatmap (left) and dotplot (right) showing the gene expression of druggable target genes uniquely identified as target genes for TF of interest in early RA. Populations, split by disease stage (early RA in orange, refractory RA in green) are plotted in columns, and genes are shown in rows. The size of the dot represents the percentage of cells expressing the markers. Gene expression is colour coded using average scaled expression values per population and per disease stage, based on a z-score distribution, ranging from low expression to high expression. F) Same as E) but for refractory RA.

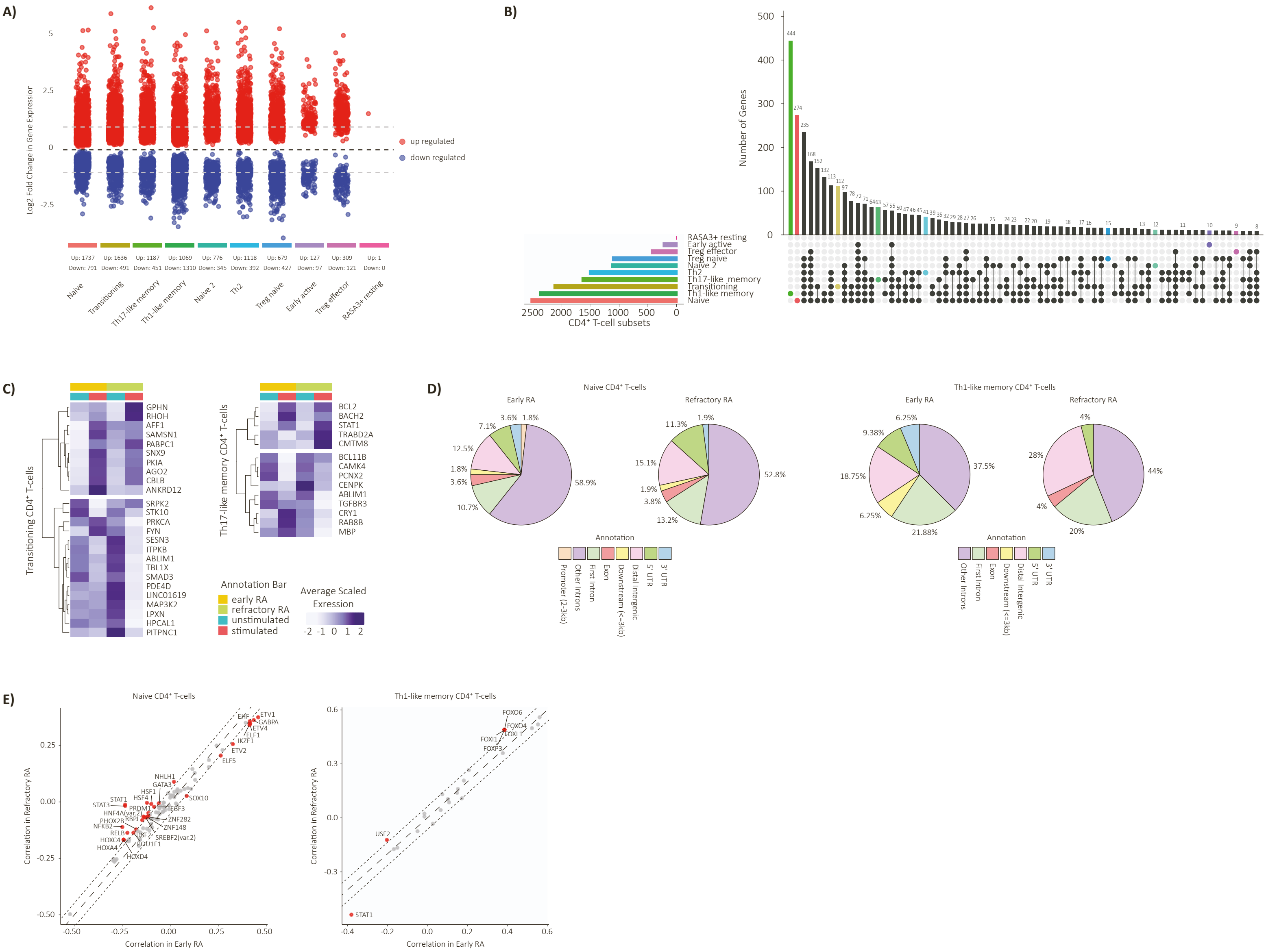

Fig. S6: Extended analysis of differential responses to stimulation by disease stage

A) Volcano plots showing the distributions of upregulated and downregulated genes following stimulation for each population of CD4⁺ T-cells (adjusted p-value<0.05). Each point denotes a differentially expressed gene (DEGs). B) UpsetPlot showing the overlap in genes differentially responding to stimulation across CD4⁺ T-cell populations. The horizontal bars represent the total number of DEGs in each population (adjusted p-value<0.05), while the vertical bars indicate the number of genes shared between populations as defined by the connected dots below. Selected CD4⁺ T-cell populations are colour-coded corresponding to population-specific DEGs. The number of intersections was capped at 70 for visualisation. C) Heatmaps showing gene expression for genes differentially responding to stimulation (adjusted p-value<0.05) in early and refractory RA cases in transitioning (left) and Th17-like memory CD4⁺ T-cells (right). Columns show average scaled gene expression values by disease stage (yellow for early RA and green for refractory RA) and by disease stage further split by unstimulated (blue) and stimulated (red) samples. Genes are shown in rows and were clustered using *k*-means clustering. Gene expression is colour coded using average scaled expression values, based on a z-score distribution, ranging from low expression (white) to high expression (purple). D) Piecharts showing the genomic annotation of stimulation-responsive peaks based on their proximity to gene features for Naïve CD4⁺ T-cells (left) and Th1-like memory CD4⁺ T-cells (right). E) Correlation between chromVAR-derived TF activity and the interaction-defined stimulation-response gene program in Naïve CD4⁺ T-cells (left) and Th1-like memory CD4⁺ T-cells (right). The stimulation-response program was generated using Seurat's AddModuleScore() function based on genes identified from the DESeq2 interaction model (genes in Figure 4A, 4B). Correlations between TF activity and gene program scores were computed separately in eRA and rRA cells using Spearman correlation. TFs positioned along the diagonal show similar TF–program relationships between disease stages, whereas deviation from the diagonal indicates disease stage-specific differences in TF–program coupling. Highlighted TFs exceeded a 1.0 SD threshold from the diagonal.

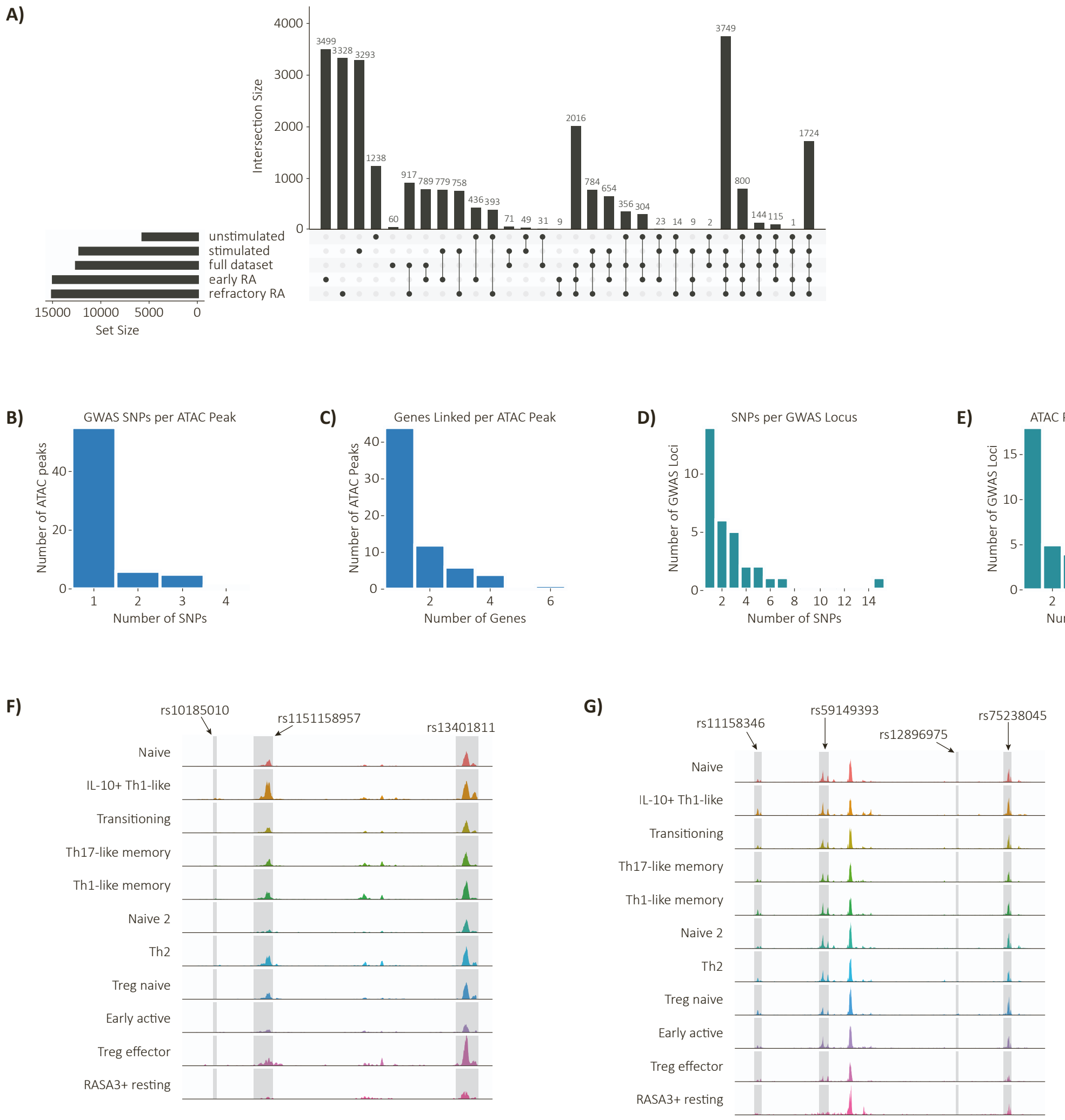

Fig. S7: Supporting CRE-GWAS SNP co-localisation analysis

A) UpsetPlot showing the overlap between all identified peak-to-gene linkage. Horizontal bars represent the total number identified within each dataset, while the vertical bars the number of unique or shared links between datasets as defined by the connected dots. B) Histogram showing the distribution of the number of RA GWAS SNPs overlapping each ATAC-seq peak. The x-axis represents the number of SNPs per peak, and the y-axis indicates the number of peaks with the corresponding SNP count. C) Histogram showing the distribution of the number of unique genes linked to each SNP containing ATAC-seq peak. The x-axis indicates the number of distinct genes associated with each peak, and the y-axis represents the number of peaks with the corresponding gene count. D) Histogram showing the distribution of the number of unique RA GWAS SNPs per RA susceptibility locus. The x-axis represents the number of distinct SNPs per GWAS locus, and the y-axis indicates the number of loci with the corresponding SNP count. E) Histogram showing the distribution of the number of unique ATAC-seq peaks overlapping each RA GWAS locus. The x-axis indicates the number of distinct peaks per locus and the y-axis represents the number of loci with the corresponding peak count. F) Chromatin accessibility landscape zoomed in on RA GWAS SNP containing peaks from Fig. 5A. G) Chromatin accessibility landscape zoomed in on RA GWAS SNP containing peaks from Fig. 5B.
